# *Salvia miltiorrhiza*-derived miRNAs suppress vascular remodeling through regulating OTUD7B/KLF4/NMHC IIA axis

**DOI:** 10.7150/thno.46911

**Published:** 2020-06-19

**Authors:** Gao-shan Yang, Bin Zheng, Yan Qin, Jing Zhou, Zhan Yang, Xin-hua Zhang, Hong-ye Zhao, Hao-jie Yang, Jin-kun Wen

**Affiliations:** 1 Department of Biochemistry and Molecular Biology, The Key Laboratory of Neural and Vascular Biology, China Administration of Education, Hebei Medical University, Shijiazhuang, China.; 2 Department of Biochemistry and Molecular Biology, Hebei University of Chinese Medicine, Shijiazhuang, China.; 3 Central Laboratory, Affiliated Hospital of Hebei University, Baoding, China.; 4 Department of Endocrine, The Second Hospital of Hebei Medical University, Shijiazhuang, China.; 5 Department of Science and Technology, The second hospital of Hebei Medical University, Shijiazhuang, China.

**Keywords:** plant-derived miRNAs, VSMC, OTUD7B, KLF4, NMHC IIA

## Abstract

**Objective:** Abnormal proliferation and migration of vascular smooth muscle cells* (*VSMCs) are essential for vascular remodeling. Natural compounds with diterpene chinone or phenolic acid structure from *Salvia miltiorrhiza,* an eminent medicinal herb widely used to treat cardiovascular diseases in China, can effectively attenuate vascular remodeling induced by vascular injury. However, it remains unknown whether *Salvia miltiorrhiza*-derived miRNAs can protect VSMCs from injury by environmental stimuli. Here, we explored the role and underlying mechanisms of *Salvia miltiorrhiza*-derived Sal-miR-1 and 3 in the regulation of VSMC migration and monocyte adhesion to VSMCs induced by thrombin.

**Methods:** A mouse model for intimal hyperplasia was established by the ligation of carotid artery and the injured carotid arteries were in situ-transfected with Sal-miR-1 and 3 using F-127 pluronic gel. The vascular protective effects of Sal-miR-1 and 3 were assessed via analysis of intimal hyperplasia with pathological morphology. VSMC migration and adhesion were analyzed by the wound healing, transwell membrane assays, and time-lapse imaging experiment. Using loss- and gain-of-function approaches, Sal-miR-1 and 3 regulation of OTUD7B/KLF4/NMHC IIA axis was investigated by using luciferase assay, co-immunoprecipitation, chromatin immunoprecipitation, western blotting, etc.

**Results:** *Salvia miltiorrhiza-*derived Sal-miR-1 and 3 can enter the mouse body after intragastric administration, and significantly suppress intimal hyperplasia induced by carotid artery ligation. In cultured VSMCs, these two miRNAs inhibit thrombin-induced the migration of VSMCs and monocyte adhesion to VSMCs. Mechanistically, Sal-miR-1 and 3 abrogate OTUD7B upregulation by thrombin via binding to the different sites of the OTUD7B 3'UTR. Most importantly, OTUD7B downregulation by Sal-miR-1 and 3 attenuates KLF4 protein levels via decreasing its deubiquitylation, whereas decreased KLF4 relieves its repression of transcription of NMHC IIA gene and thus increases NMHC IIA expression levels. Further, increased NMHC IIA represses VSMC migration and monocyte adhesion to VSMCs via maintaining the contractile phenotype of VSMCs.

**Conclusions:** Our studies not only found the novel bioactive components from *Salvia miltiorrhiza* but also clarified the molecular mechanism underlying Sal-miR-1 and 3 inhibition of VSMC migration and monocyte adhesion to VSMCs. These results add important knowledge to the pharmacological actions and bioactive components of *Salvia miltiorrhiza*. Sal-miR-1 and 3-regulated OTUD7B/KLF4/NMHC IIA axis may represent a therapeutic target for vascular remodeling.

## Introduction

In response to injury, vascular smooth muscle cells (VSMCs) change their phenotype from a contractile to a synthetic type, accompanied by the downregulation of VSMC marker genes and upregulation of genes related to inflammation, proliferation and migration 1-3. The proliferation, migration and adhesion of VSMCs play a pivotal role in the pathogenesis of vascular remodeling induced by various stimuli. Previous studies suggested that VSMC phenotypes are modulated by many factors, such as platelet-derived growth factor-BB (PDGF-BB) 4, transforming growth factor-β (TGF-β) 5, thrombin 6, angiotensin II (Ang II) 7, and so on.

Emerging evidences have shown that, besides decreased expression of VSMC marker genes and increased expression of inflammation, proliferation, and migration-related genes, the expression of non-muscle myosin heavy chain IIA (NMHC IIA), a major member of the type II non-muscle myosin family encoded by the MYH9 gene, is also markedly altered during cell proliferation, migration and phenotypic switching 8-10. Specifically, deletion of NMHC IIA results in disintegration of actin myofilament 11 and inhibits F-actin assembly into stress fibers 12, affecting cell morphology and migration 13. Krüppel-like factor 4 (KLF4) shows developmental and pathological implications in the vasculature 3. KLF4 not only regulates the expression of VSMC marker genes involved in F-actin formation and reorganization, such as SM α-actin and smooth muscle protein 22α (SM22α), but also transactivates the expression of migration-related genes 14. However, the actual relationship between KLF4 and NMHC IIA expression in the regulation of VSMC migration and adhesion remains to be investigated.

Recently, we found that salvianolic acid B, a water-soluble compound extracted from *Salvia miltiorrhiza* widely used to treat cardiovascular diseases for hundreds of years in China, can effectively suppresses Ang II-induced VSMC proliferation *in vitro* and intimal hyperplasia *in vivo* by downregulating miR-146a expression 15. The chemical constituents of *Salvia miltiorrhiza* have been well identified, including more than 30 lipophilic compounds that have a diterpene chinone structure and more than 50 hydrophilic compounds that mainly have a phenolic acid structure 16. *Salvia miltiorrhiza* exerts cardiovascular protection through multiple mechanisms including anti-oxidation, anti-thrombosis, anti-inflammation, and leukocyte-endothelial adhesion regulation 17, 18. In recent years, plant-derived miRNAs were found to be present in human serum and tissues and are capable of regulating mammalian gene expression, providing an evidence of cross-kingdom regulation by miRNAs 19-21. Besides the above-mentioned active constituents in *Salvia miltiorrhiza*, whether do *Salvia miltiorrhiza* miRNAs also have the cross-kingdom regulation of mammalian VSMCs? Since plant miRNAs are 2′-O-methyl modified on their terminal nucleotide 22, and are more stable than mammalian miRNAs, it is of great significance for the development of novel vasoprotective agents to identify *Salvia miltiorrhiza*-derived miRNAs and to investigate the molecular mechanism underlying the regulation of migration and adhesion of VSMCs by them.

In this study, we investigated whether and how *Salvia miltiorrhiza*-derived Sal-miR-1 and 3 regulate the migration and monocyte adhesion to VSMCs induced by thrombin, as well as attenuate vascular remodeling induced by vascular injury through targeting OTUD7B/KLF4/NMHC IIA regulatory axis.

## Materials and Methods

### High-throughput sequencing and bioinformatics approaches

High-throughput sequencing and bioinformatics for Salvia miltiorrhiza were performed in Novegene in Beijing, China. A total amount of 3 μg total RNA was used for input material for plant miRNA database. Sequencing database was generated using NEBNext® Multiplex Small RNA Library Prep Set for Illumina® (NEB, USA.) following manufacturer's recommendations and index codes were added to attribute sequences of each sample. The small RNA tags were mapped to the reference sequence by Bowtie without mismatch to analyze their expression, and the characteristics of hairpin structure of miRNA precursor can be used to predict novel miRNA. At the same time, custom scripts were used to obtain the identified miRNA counts as well as base bias on the first position with certain length and on each position of all identified miRNA, respectively. High-throughput sequencing and bioinformatics for *Salvia miltiorrhiza* were independently repeated and verified by qRT-PCR.

### Animal model and treatment

Animal housing, handling and all procedures were approved by the Institutional Animal Care and Use Committee of Hebei Medical University (approval ID: HebMU 20080026) and all efforts were made to minimize suffering. Initial hyperplasia model induced by carotid artery ligation was generated as described previously 23. Briefly, 6-8-week-old male wild-type (WT) C57BL/6 mice were anesthetized with 2% isoflurane gas. The left common carotid artery was dissected and ligated near the carotid bifurcation using a 6-0 suture. In unligated animals, the suture was passed under the exposed left carotid artery but not tightened. *Salvia miltiorrhiza* extract was administered by intragastric administration at a dose of 1 g kg^-1^ day^-1^ (n=9) beginning 3 days before ligation and continuing for 21 days thereafter. For the Sal-miR-1 and 3 *in situ* transfection, synthetic Sal-miR-1+3 (1 OD, n=6) or control miRNA (1 OD, n=6) were added into the 150 μl of 20% F-127 pluronic gel (Sigma) at 4 °C for 2 h. Immediately after ligation, 150 μl pluronic gel containing these miRNAs was applied to the exposed adventitial surface of an approximately 5 mm segment of the ligated carotid artery. At 21 days after surgery, all animals were anesthetized and perfused with cold PBS, and tissues were harvested for miRNA, morphology or histological analysis.

### Morphology analysis

The injured carotid arteries by ligation were anatomically localized and cut as described previously 23. In brief, the common carotid arteries were approximately 9 mm long, of which the proximal and distal 2 mm were discarded and the remaining portion (approximately 5 mm) was cut in half. The two segments were embedded in paraffin, and serial sections (4 μm thick) were cut for analysis by immunofluorescent staining for SM α-actin (mouse monoclonal, 1:50, sc-130617, Santa Cruz), OTUD7B (rabbit polyclonal, 1:50, 16605-1-AP, Proteintech) or VCAM-1 (rabbit monoclonal, 1:250, ab134047, Abcam) as well as by hematoxylin-eosin or Elastic-van Gieson staining for morphometry. Five or more sections spanning most of the vessel segment were analyzed for morphometry. The neointimal area and intima-to-media ratio were calculated using Image-Pro Plus Analyzer (version 5.1) software (Media Cybernetics, Silver Spring, MD) in a blinded manner.

### Cell culture

Mouse aortic vascular smooth muscle cells (VSMC) (ATCC, No. CRL-2797™) were routinely cultured in low-glucose Dulbecco's modified Eagle's medium (DMEM, Gibco Life Technologies, 31600-034, Rockville, MD) containing 100 units/mL of penicillin, 0.1 mg/mL of streptomycin and 10% fetal bovine serum (GEMINI, 900-108, USA), in a humidified incubator at 37 °C with 5% CO_2_. The growth medium was replaced every 2 days, and the cells were passaged every 4 days at a ratio of 1 to 4 upon 80% confluence. Prior to stimulation with thrombin (Sigma, T4393), cells were incubated in serum-free medium for 24 h.

### Isolation of RNA and real-time PCR

Total RNA was extracted from *Salvia miltiorrhiza* using the plant RNA kit (OMEGA, R6827-01) according to the manufacturer's instructions. A 100 μl mixture consisting of 20 μg of small RNA fraction and 10 mM NaIO_4_ (Thermo Scientific, 20504) was incubated at 0 °C for 40 min in dark. The oxidized RNA was precipitated twice by ethanol, rinsed once with 80% ethanol, air-dried, dissolved in ddH_2_O, and then subjected to Solexa sequencing or qRT-PCR assay. For qRT-PCR assay, reverse transcription and real-time PCR were performed using the plant microRNA Reverse Transcription Kit and Universal Master MixII (Qiagen, 69106) with specific primers for Sal-miR-1, Sal-miR-3 and internal control RNU6b (U6) according to the manufacturer's protocol.

Total RNA was extracted from the serum or tissues with TRIzol reagent (Qiagen, 10296-028) according to the manufacturer's instructions, and miRNeasy Mini Kit was used to extract tissue RNA, miRNeasy Serum/Plasma Kit was used to extract RNA from serum. The quality of the RNA was measured using the NanoDrop 2000 Spectrophotometer (Thermo Scientific). Reverse transcription and qRT-PCR were run on ABI 7500 FAST system (Life Technologies) using the miRNA Detection Kits by Genepharma (Shanghai, China) or DNeasy Plant Mini Kit (Qiagen, 69106) according to the manufacturer's protocol. U6 was used as internal control.

Total RNA was extracted from cultured VSMCs using the Trizol (InvitrogenTM) according to the manufacturer's instructions. The quality of the RNA was determined using a Biospectrometer (Eppendorf). cDNA was synthesized using an M-MLV First Strand Kit (Life Technologies), and real-time PCR analysis was done with the ABI 7500 FAST system, using the Platinum SYBR Green qPCR SuperMix UDG Kit (Invitrogen), according to the manufacturer's instructions. As an internal control, 18s primers were used for RNA template normalization. All PCRs were performed in triplicate. Relative amount of transcripts was calculated using the 2^-ΔΔCt^ formula. All the sequences of primers or miRNAs used are listed as Table 1.

### Oxidation of microRNAs with sodium periodate

Periodate oxidation of microRNA was performed as previously described with slight modifications 19, 22, 24. Briefly, total RNA was extracted from *Salvia miltiorrhiza*, mouse serum or tissues using the Trizol Reagent according to the manufacturer's instructions. A 100 μl mixture consisting of 20 μg of small RNA fraction and 10 mM NaIO_4_ (Thermo Scientific, 20504) was incubated at 0 °C for 40 min in dark. The oxidized RNA was precipitated twice by ethanol, rinsed once with 80% ethanol, air-dried, dissolved in ddH_2_O, and then subjected to Solexa sequencing or qRT-PCR assay. The qRT-PCR assay was conducted using the miScript PCR system (Qiagen, 218161) according to the manufacturer's instructions.

### Western blot analysis

Proteins were isolated from cultured VSMCs with lysis buffer (1% Triton X-100, 150 mM NaCl, 10 mM Tris-HCl, pH 7.4, 1 mM EDTA, 1 mM EGTA, pH 8.0, 0.2 mM Na_3_VO_4_, 0.2 mM phenylmethylsulfonyl fluoride, and 0.5% NP-40). Equal amounts of protein were separated on 10% SDS-PAGE, and electrotransferred to a PVDF membrane (Millipore). Membranes were blocked with 5% milk in TTBS for 2 h at room temperature and incubated with primary antibodies overnight at 4 °C. Antibodies that were used are as follows: anti-OTUD7B (rabbit polyclonal, 1:1000, 16605-1-AP, Proteintech), anti-VCAM-1 (rabbit monoclonal, 1:1000, ab134047, Abcam), anti-ICAM-1 (rabbit polyclonal, 1:1000, 10020-1-AP, Proteintech), anti-SMα-actin (rabbit polyclonal, 1:1000, 14395-1-AP, Proteintech) anti-KLF4 (rabbit polyclonal, 1:1000, ab106629, Abcam), anti-SM22α (rabbit polyclonal, 1:1000, 10493-1-AP, Proteintech), anti-NMHC IIA (rabbit polyclonal, 1:1000, 11128-1-AP, Proteintech), anti-KLF3 (mouse monoclonal, 1:1000, sc-514500, Santa Cruz), anti-KLF10 (rabbit polyclonal, 1:1000, ab73537, Abcam), anti-Calponin (rabbit polyclonal, 1:1000, 13938-1-AP, Proteintech), anti-SMMHC (rabbit polyclonal, 1:1000, 21404-1-AP, Proteintech) and anti-β-actin (mouse monoclonal, 1:1000, sc-47778, Santa Cruz). Then, membranes were washed with TTBS, and incubated with secondary antibodies conjugated with HRP (1:5000, Rockland, KOA0136) at room temperature for 1 h. Protein blots were treated with the Immobilon™ Western (Millipore), and detected by ECL (enhanced chemiluminescence) Fuazon Fx (Vilber Lourmat). Images were captured and processed by FusionCapt AdvanceF×5 software (Vilber Lourmat).

### Cell transfection

The VSMCs were transfected using Lipofectamine 2000 (Invitrogen) according to the manufacturer's protocol. Small interfering RNAs (siRNAs) specific for mouse OTUD7B (Gene ID: 229,603), KLF4 (Gene ID: 16,600), NMHC IIA sequences (Gene ID: 17,886) and non-specific siRNA (si-Ctl) were designed and synthesized by Genepharma. The siRNA sequences used in these studies were as follows: KLF4 siRNA: 5′-CUA ACC GUU GGC GUG AGG AAC TT-3′; 5′-GUU CCU CAC GCC AAC GGU UAG TT-3′; OTUD7B siRNA: 5′-GCA GUG GUA CGG AGA CAU UTT -3′; 5′-AAU GUC UCC GUA CCA CUG CTT -3′; NMHC IIA siRNA: 5′-UAU AAG GGU UGA CCA CTT -3′; 5′-GUG GUC AUC AAC CCU UAU ATT-3′; si-Ctl: 5′-UUC UCC GAA CGU GUC ACG UTT-3′; 5′-ACG UGA CAC GUU CGG AGA ATT-3′. After 24 h of transfection, the VSMCs were incubated with 2% FCS and stimulated with thrombin (Sigma, T4393) for 24 h. The cells were then harvested and lysed for qRT-PCR or Western blot analysis.

### Immunofluorescence staining

Immunofluorescence staining was performed on 4 μm paraffin cross-sections from the carotid arteries. VSMCs were prepared as described above. The sections were deparaffinized with xylene and rehydrated, cells were fixed by 4% paraformaldehyde and then were permeabilized by incubation with 0.1% Triton X-100 in PBS. Non-specific sites were blocked by incubation in 10% normal goat serum (710027, KPL, USA) for 1 h. Then the sections or cells were incubated with primary antibodies at 4 °C overnight. The primary antibodies were anti-SMα-actin (mouse monoclonal 1:100, sc-130617, Santa Cruz), VCAM-1 (rabbit monoclonal, 1:100, ab134047, Abcam), anti-OTUD7B (rabbit polyclonal, 1:50, 16605-1-AP, Proteintech), anti-NMHC IIA (rabbit polyclonal, 1:50, 11128-1-AP, Proteintech), anti-NMHC IIA (mouse monoclonal, 1:50, 60233-1-Ig, Proteintech). Secondary antibodies were rhodamine-labeled antibody to rabbit IgG (1:50, 031506, KPL, USA) and fluorescein-labeled antibody to mouse IgG (1:50, 021815, KPL, USA), or rhodamine-labeled antibody to mouse IgG (1:50, 031806, KPL, USA) and fluorescein-labeled antibody to rabbit IgG (1:50, 021516, KPL, USA). Nuclei were stained with DAPI (1:50, 157574, MB biomedical) in each experiment. Images were captured by confocal microscopy (DM6000 CFS, Leica) and processed by LAS AF software.

### Phalloidin staining for actin stress fibers

VSMCs grown on coverslips were fixed in 4% paraformaldehyde and then were permeabilized with 0.1% Triton X-100 in PBS at room temperature for 10 min, followed by tetramethyl rhodamine isothiocyanate (TRITC)-phalloidin (Sigma) staining for 30 min in the dark. Nuclei were stained with DAPI (1:50, 157574, MB biomedical) to visualize nuclear localization. Images were captured by confocal microscopy (Leica) and processed by LAS AF software.

### Chromatin immunoprecipitation assays (ChIP)

The VSMCs were fixed in 1% formaldehyde for 10 min to cross link proteins with DNA. The cross-linked chromatin was then prepared and sonicated to an average size of 400-600 bp. The DNA fragments were immunoprecipitated overnight at 4 ℃ with the antibodies against KLF4 (rabbit polyclonal, ab106629, Abcam) and normal rabbit IgG (Santa Cruz) antibodies. After reversal of cross-linking, the precipitated DNA was recovered via phenol/chloroform extraction, and the KLF4 binding site (the region between -1841 to +42 bp) was examined by qPCR with the primers listed on the Table 2 for ChIP, and a negative control region upstream of the KLF4 promoter (the region between -1558 to -1353 bp) was also amplified. All experiments were replicated at least three times.

### Co-immunoprecipitation assay

Co-immunoprecipitation was performed as follows. The tissue extracts or cell lysates were immunoprecipitated with 3 μg of anti-OTUD7B (rabbit polyclonal, 16605-1-AP, Proteintech), anti-KLF4 (rabbit polyclonal, ab106629, Abcam), anti-NMHC IIA (rabbit polyclonal, 11128-1-AP, Proteintech) or anti-ubiquitin (rabbit polyclonal, PTM-1106, PTM BIO), respectively, for 1 h at 4 °C, then incubated with protein A-agarose overnight at 4 °C. The complexes of Protein A-agarose-antibody were collected by centrifugation at 12,000 rpm for 2 min at 4 °C, and washed 4 times with 500 μl of IPH buffer (50 mM Tris-HCl, pH 8.0, 150 mM NaCl, 5 mM EDTA, 0.5% NP-40, and 0.1 mM phenylmethylsulfonyl fluoride) for 20 min at 4 °C. The bound proteins were resolved using SDS-polyacrylamide gel followed by Western blotting using anti-OTUD7B (rabbit polyclonal, 1:1000, 16605-1-AP, Proteintech), anti-KLF4 (rabbit polyclonal, 1:1000, ab106629, Abcam), anti-NMHC IIA (rabbit polyclonal, 1:1000, 11128-1-AP, Proteintech) or anti-ubiquitin (rabbit polyclonal, 1:2000, PTM-1106, PTM BIO) antibodies.

### Immunohistochemistry

Paraffin cross-sections were deparaffinized with xylene and rehydrated in a graded ethanol series, and endogenous peroxidase activity was inhibited by incubating with 3% H_2_O_2_. Sections were blocked with 5% goat serum in PBS and incubated overnight at 4 °C with primary antibodies. After a PBS wash, sections were incubated with secondary antibody at 37 °C for 30 min. Immunohistochemical staining was visualized using a diaminobenzidine kit (Zhongshan Golden bridge Biotechnology, Beijing, China) according to the manufacturer's instructions. Sections were counterstained with hematoxylin to visualize nuclei. Primary antibodies included anti-VCAM-1 (rabbit monoclonal, 1:250, ab134047, Abcam).

### Luciferase assay

The 3' untranslated region (UTR) sequences of OTUD7B containing wild-type or mutant forms of the Sal-miR-1, Sal-miR-3 or Sal-miR-1 and 3 target site were inserted into the *Xho1* and *Sal1* digested-pmir-GLO Dual-Luciferase miRNA Target Expression Vector (Promega Corp., Madison, WI, USA), generating wild-type or mutant OTUD7B 3' UTR reporter vector. For luciferase assays, 3×10^4^ HEK293A cells/well were seeded in a 24-well plate and grown for 24 h and then transfected with a 100 ng wild-type or mutant OTUD7B 3' UTR reporter vector, Sal-miR-1, Sal-miR-3 or Sal-miR-1 and 3, and pTK-RL (Renilla luciferase reporter) plasmid using Lipofectamine 2000 reagent (Invitrogen) according to the manufacturer's instructions. Luciferase assays were performed using Dual-Glo Luciferase Assay System (Promega, Madison, WI) 24 h after transfection. Specific promoter activity was expressed as the relative activity ratio of firefly luciferase to Renilla luciferase. All promoter constructs were evaluated in at least 3 separate wells per experiment.

### Cell migration assays

For wound healing, VSMCs were grown to 80-100% confluence, and then the cell monolayer was scratched using a sterile micropipette tip. After washing with PBS, fresh medium was added. The images of the wounded area were captured immediately 0 and 24 h after scratch to monitor VSMC migration into the wounded area. The migratory abilities were quantified by measuring the total number of cells in the scratched regions.

For cell invasion assays, Boyden Chamber CytoSelect Cell Migration Assay kit with polycarbonate membrane inserts was used (8 μm pore size; Cell Biolabs Inc.). Briefly, VSMCs were added to the upper chamber at 2×10^4^ cells/well. A medium containing thrombin was added to the lower chamber. After 12 h incubation, non-migratory cells on the upper membrane surface were removed, and the cells that traversed and spread on the lower membrane surface were fixed with methanol and stained with hemotoxylin and eosin (Modified solution, Sigma). The number of migratory cells per membrane was counted. Four random fields in each filter were examined. Each experiment was performed in triplicate, and migration was expressed as the mean ± SEM of total cells counted per field.

### Cell adhesion assays

Primary bone marrow-derived macrophages were obtained from femoral bone marrow suspensions of C57BL/6 mice and plated at 3×10^6^ cells/mL and differentiated for 7 d in the DMEM medium with 10 % FBS and 5×10^-3^ mg/mL murine G colony-stimulating factor (Pepro Tech, no. A2714). Mouse RAW 264.7 cells were cultured in DMEM medium supplemented with 10 % FBS and 1% penicillin/streptomycin (100 U/100 mg) solution (Life Technologies, Grand Island, NY).

For macrophage adhesion to VSMCs, VSMCs grown on glass coverslips in 6-well plates were transfected with Sal-miR-1 and 3 for 6 h, and then treated with thrombin for 24 h. Then the RAW264.7 cells or primary bone marrow-derived macrophages were added to each hole, and incubated at 37 °C for 30 min. The coverslips were washed with fresh serum-free medium 3 times for 15 min each, and the non-adherent macrophages were washed out. The coverslips were collected, and 4% paraformaldehyde was added and fixed for 10 min. Immunofluorescence staining for VSMCs using anti-SM α-actin (rabbit polyclonal, 1:100, 14395-1-AP, Proteintech), macrophage using anti-MAC-2 (mouse monoclonal, 1:50, 60207-1-Ig, Proteintech) was performed. At least five random fields in each coverslip were observed, and the number of cells was counted.

### Time-lapse experiments

VSMCs were transfected with Sal-miR-1 and 3 or si-NMHC IIA and treated with or without thrombin. Time-lapse images were acquired using a Leica AF6000 inverted microscope equipped with an on-stage incubation chamber that maintained the temperature at 37 °C and the CO_2_ concentration at 5% at all times. VSMCs were imaged via a 40× objective, and repetitive imaging was performed every 15 min for up to 12 h. Quantification of cell shapes and determination of the location were performed on an image analysis by using NIH Image J software.

### Statistical analysis

All analyses were performed using the SPSS 21.0 software and GraphPad Prism 7 software (GraphPad Software, La Jolla, CA). Data are expressed as the means ± standard error of the mean (SEM). Two groups were analyzed by Student's t-test. Differences among groups were analyzed with one-way analysis of variance (ANOVA). For all analyses, a value of *P* < 0.05 was considered significant. All of the presented data were repeated at least three independent experiments.

## Results

### *Salvia miltiorrhiza*-derived Sal-miR-1 and 3 suppress intimal hyperplasia induced by carotid artery ligation in mice

**Figure 1:**
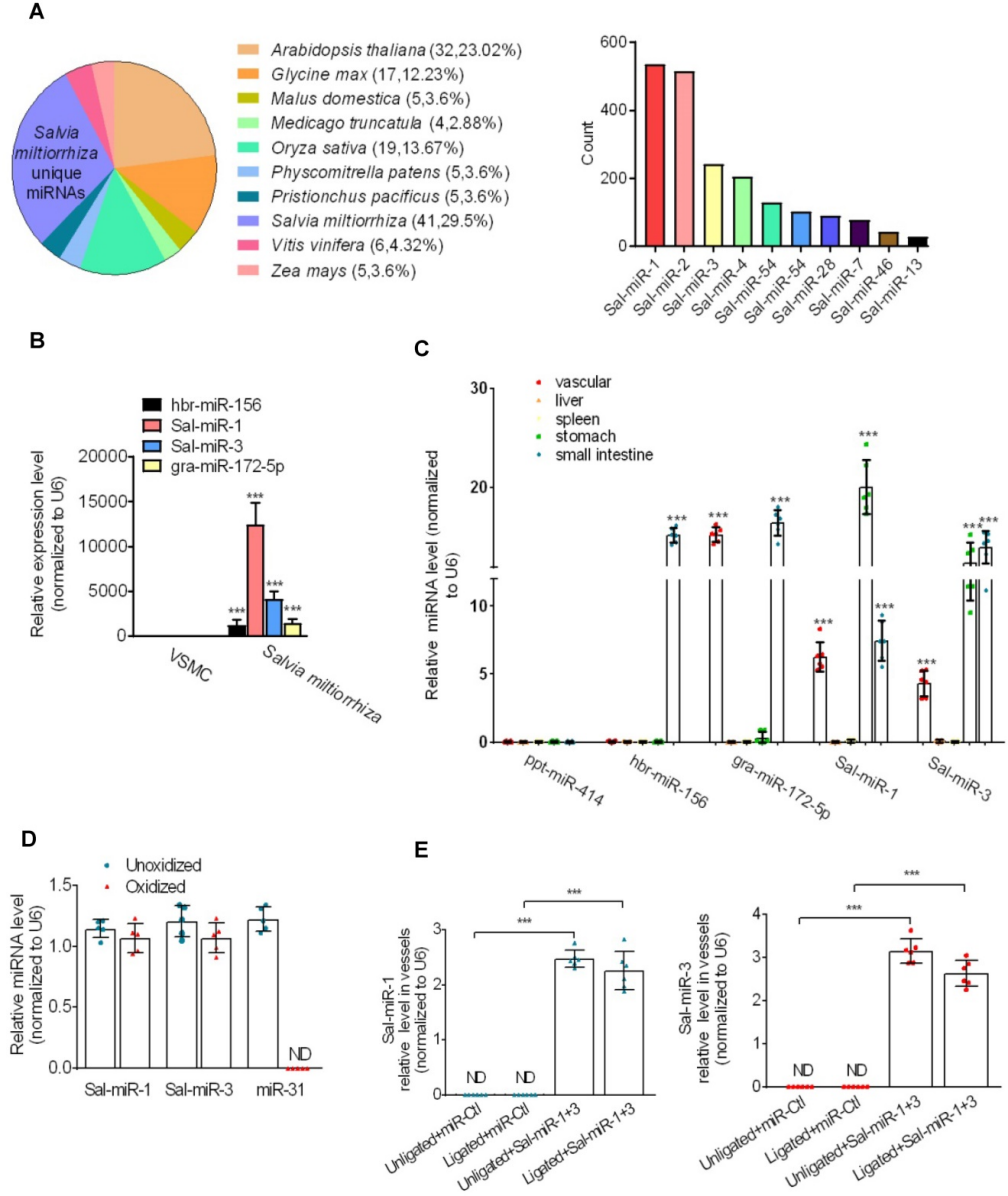

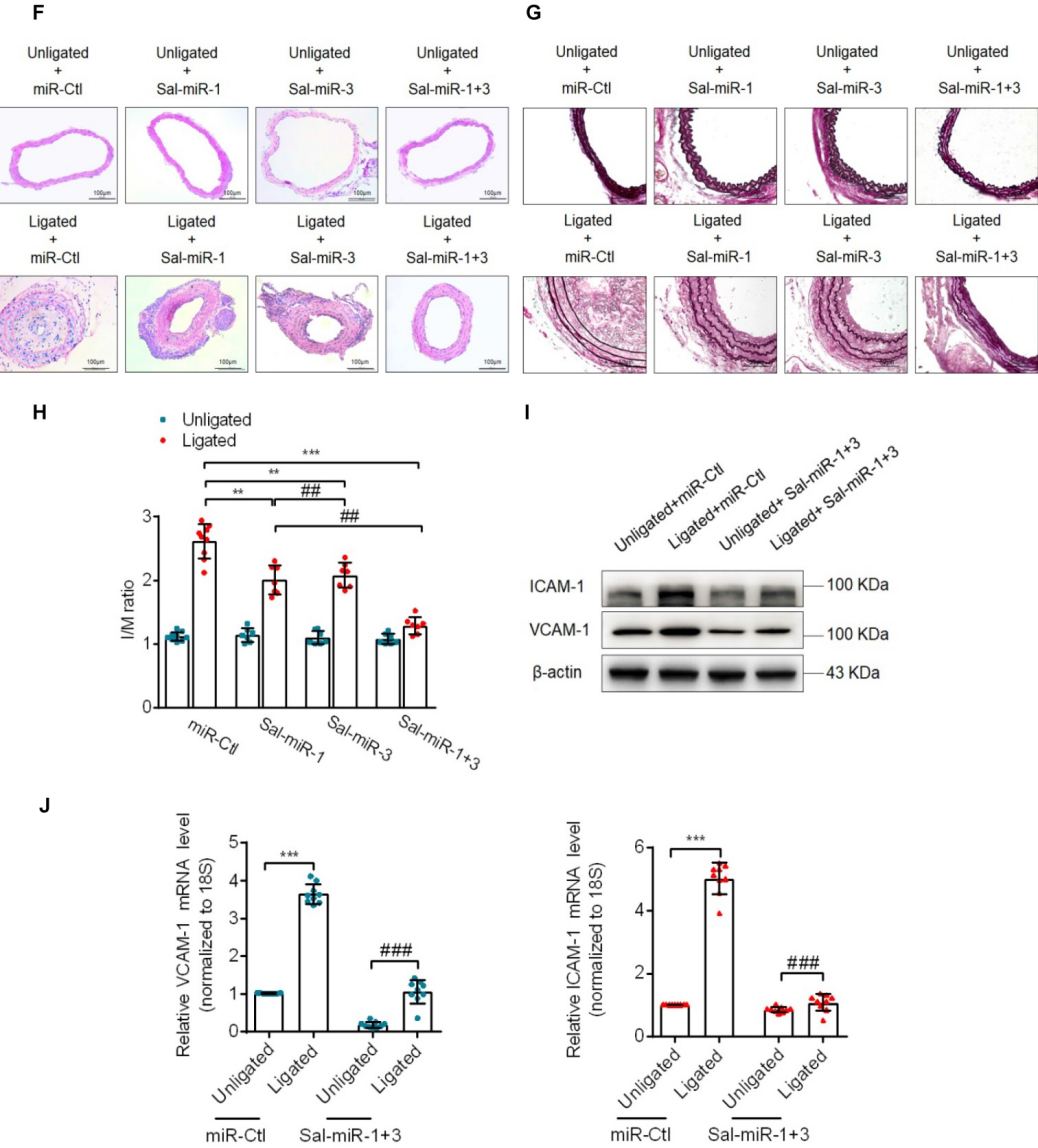
***Salvia miltiorrhiza*-derived Sal-miR-1 and 3 may enter the mouse body after intragastric administration and inhibits intimal hyperplasia.** (**A**) High-throughput sequencing and bioinformatics analysis show miRNAs extracted from *Salvia miltiorrhiza.* (**B**) Relative expression level of *Salvia miltiorrhiza*-derived Sal-miR-1, Sal-miR-3, hbr-miR-156, and gra-miR-172-5p was examined by qRT-PCR. ***P<0.001 versus VSMCs. (**C**)* Salvia miltiorrhiza* was administered to mice by gavage once a day for 3 weeks, qRT-PCR detected the expression of *Salvia miltiorrhiza*-derived miRNAs in the indicated tissues of C57BL/6J mice. Relative expression level of five selected miRNAs were detected by qRT-PCR (n=9). (**D**) miRNAs isolated from mouse artery tissues were treated with/without sodium periodate, and Sal-miR-1, Sal-miR-3 and miR-31 were detected by qRT-PCR (n=5). (**E**) The injured carotid arteries were in situ*-*transfected with Sal-miR-1+3, qRT-PCR detected Sal-miR-1 and 3 expression (n=6 mice per group). (**F-G**) Representative hematoxylin and eosin or Elastic-van Gieson staining of cross-sections from unligated and ligated carotid arteries transfected with miR-Ctl (n=9), Sal-miR-1 (n=7), Sal-miR-3 (n=7) or Sal-miR-1+3 (n=7). Scale bars=100 µm or 50 µm. (**H**) The ratio of intimal/medial area (I/M ratio) was measured in the injured carotid arteries treated as in (F-G). (**I**) Western blot analysis detected ICAM-1 and VCAM-1 expression in the unligated and ligated arteries treated or not with Sal-miR-1+3 (n=9). (**J**) The carotid arteries were treated as in (I), and the expression of ICAM-1 and VCAM-1 mRNA was detected by qRT-PCR (n=9). Bar graphs show mean±SEM from 3 independent experiments (n=3). ND, Not Detected. Student's t-test or one-way ANOVA test: **P<0.01, ***P<0.001,^ ##^*P*<0.01,^ ###^*P*<0.001 versus the corresponding control.

To identify *Salvia miltiorrhiza*-derived miRNAs with vasoprotective properties, we first used the Illumina/Solexa deep-sequencing technology to sequence all the short RNA fragments extracted from *Salvia miltiorrhiza* and found that *Salvia miltiorrhiza* contained multiple unique miRNAs. Among them, Sal-miR-1 and 3 had a higher copy number than other miRNAs (Figure 1A), and Solexa sequencing results were further confirmed by qRT-PCR, showing that Sal-miR-1 and 3 were highly expressed in *Salvia miltiorrhiza* (Figure 1B). Next, we assessed the absorption of* Salvia miltiorrhiza*-derived miRNAs after *Salvia miltiorrhiza* extract was intragastrically administered to mice. The results showed that high levels of Sal-miR-1 and 3 were present in mouse blood vessel, stomach and small intestine, but not liver and spleen (Figure 1C). Also, high levels of circulating Sal-miR-1 and 3 were detected in patients administrated with oral *Salvia miltiorrhiza* (Figure S1A). These results suggest that *Salvia miltiorrhiza*-derived miRNAs may enter the mouse and human body after exogenous administration. Considering that Sal-miR-1 and 3 were more abundant in *Salvia miltiorrhiza,* and they were highly enriched in the vasculature after *Salvia miltiorrhiza* extract was administered to mice, we made a further study on Sal-miR-1 and 3. Further, we used sodium periodate oxidation to confirm that the Sal-miR-1 and 3 in mouse serum were resistant to oxidizing agent due to the 2'-O-methylation on their 3'-terminal ribose, which is characteristic of plant-derived miRNAs 22, 25. In contrast, mammalian miR-31 was oxidized due to the lack of this protection (Figure 1D).

Because it is well known that *Salvia miltiorrhiza* exerts multiple biological activities, such as anti-oxidant, anti-inflammatory and anti-proliferation in different tissues 16, we investigated whether Sal-miR-1 and 3 suppress intimal hyperplasia of carotid artery induced by ligation through anti-inflammation and anti-proliferation. Following carotid artery ligation, Pluronic F-127 gel solution containing a miR-Ctl (control, 1 OD) or synthetic Sal-miR-1 (1 OD) and Sal-miR-3 (1 OD) was applied to the exposed adventitial surface of an approximately 5 mm segment of the ligated carotid artery. The level of Sal-miR-1 and 3 in the carotid arteries transfected with these two miRNAs increased by >2-fold relative to that of mice treated with miR-Ctl (Figure 1E). Further, ligation-induced intimal hyperplasia in miR-Ctl (control)-transfected carotid artery was established by morphometric analysis. Importantly, we confirmed that transfecting vessels with Sal-miR-1 or Sal-miR-3 alone or together significantly decreased neointimal hyperplasia, as shown by hematoxylin and eosin or Elastic-van Gieson staining (Figure 1F-G) as well as attenuated the ratio of intimal/medial area (I/M ratio) (Figure 1H). We also examined the effect of intragastric administration of Sal-miR-1 and 3 on vascular remodeling induced by carotid ligation and found that intragastric administration of Sal-miR-1 and 3 could suppress ligation-induced neointimal formation, but the inhibitory effect was lower than that of *in situ* transfection of Sal-miRNAs (Figure S1B-C). These findings suggest that Sal-miR-1 or Sal-miR-3 alone or together can significantly suppress the neointimal hyperplasia induced by vascular injury. Because the inhibitory effect of Sal-miR-1 and 3 on neointimal formation was more significant than one of them alone*,* we chose Sal-miR-1 and 3 to investigate the mechanism(s) by which Sal-miR-1 and 3 suppress neointimal hyperplasia in all subsequent experiments.

Because VSMCs in the injured arteries are known to express adhesion molecules 26, which are required for inflammation induced by vascular injury, we sought to examine whether Sal-miR-1 and 3 affect ICAM-1 and VCAM-1 expression after exogenous administration. The results showed that carotid artery ligation-induced vascular injury significantly upregulated the protein levels of both adhesion molecules compared with the unligated vessels, whereas Sal-miR-1 and 3 attenuated ICAM-1 and VCAM-1 protein expression (Figure 1I). The mRNA expression level of these two adhesion molecules was consistent, as shown by qRT-PCR (Figure 1J). The similar results were also obtained by immunofluorescence and histochemical staining of VCAM-1 (Figure S1D-E). These results indicated that Sal-miR-1 and 3 significantly inhibit vascular injury-induced intimal hyperplasia and the expression of adhesion molecules.

### Sal-miR-1 and 3 attenuate thrombin-induced VSMC migration and monocyte adhesion to VSMCs

**Figure 2:**
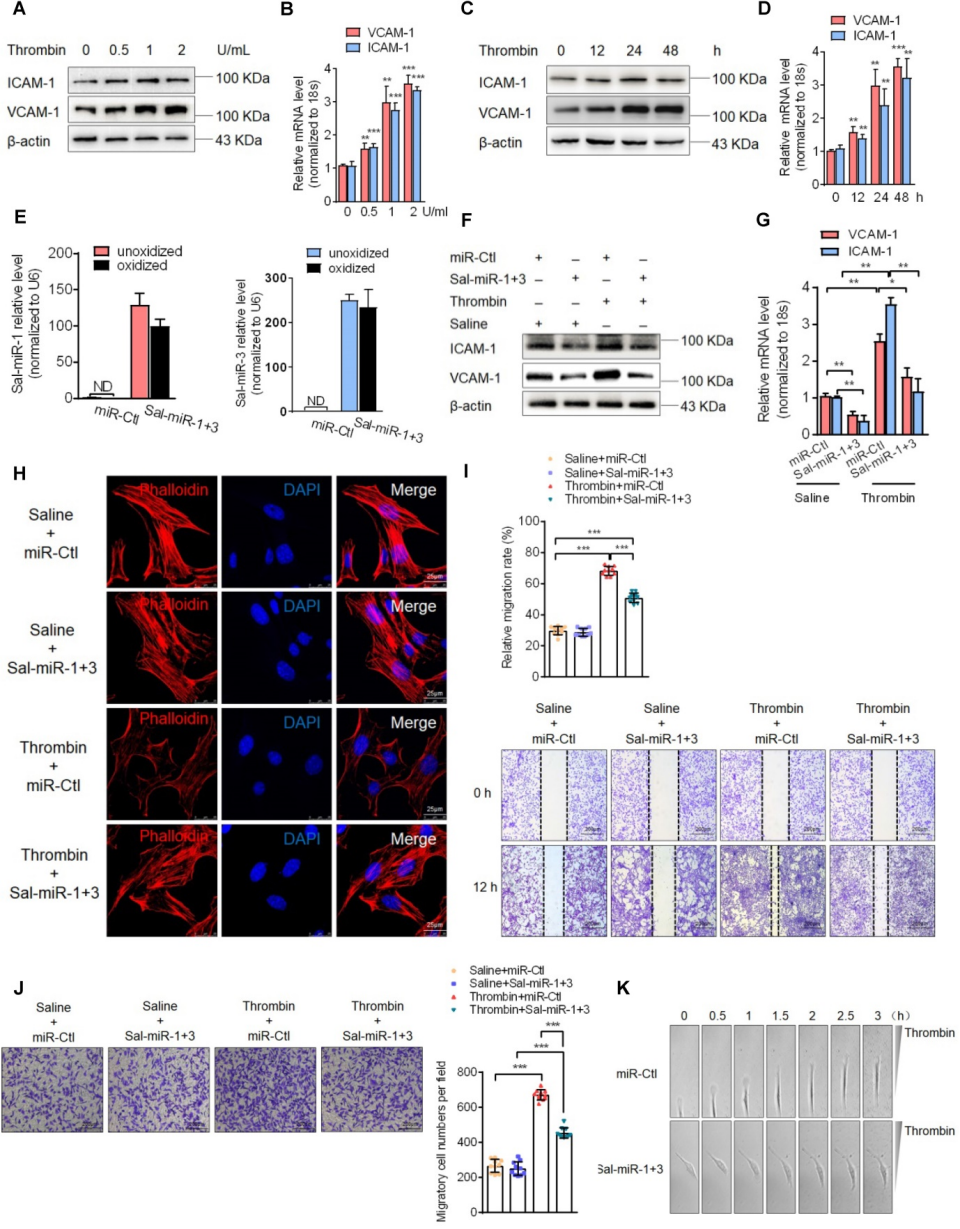

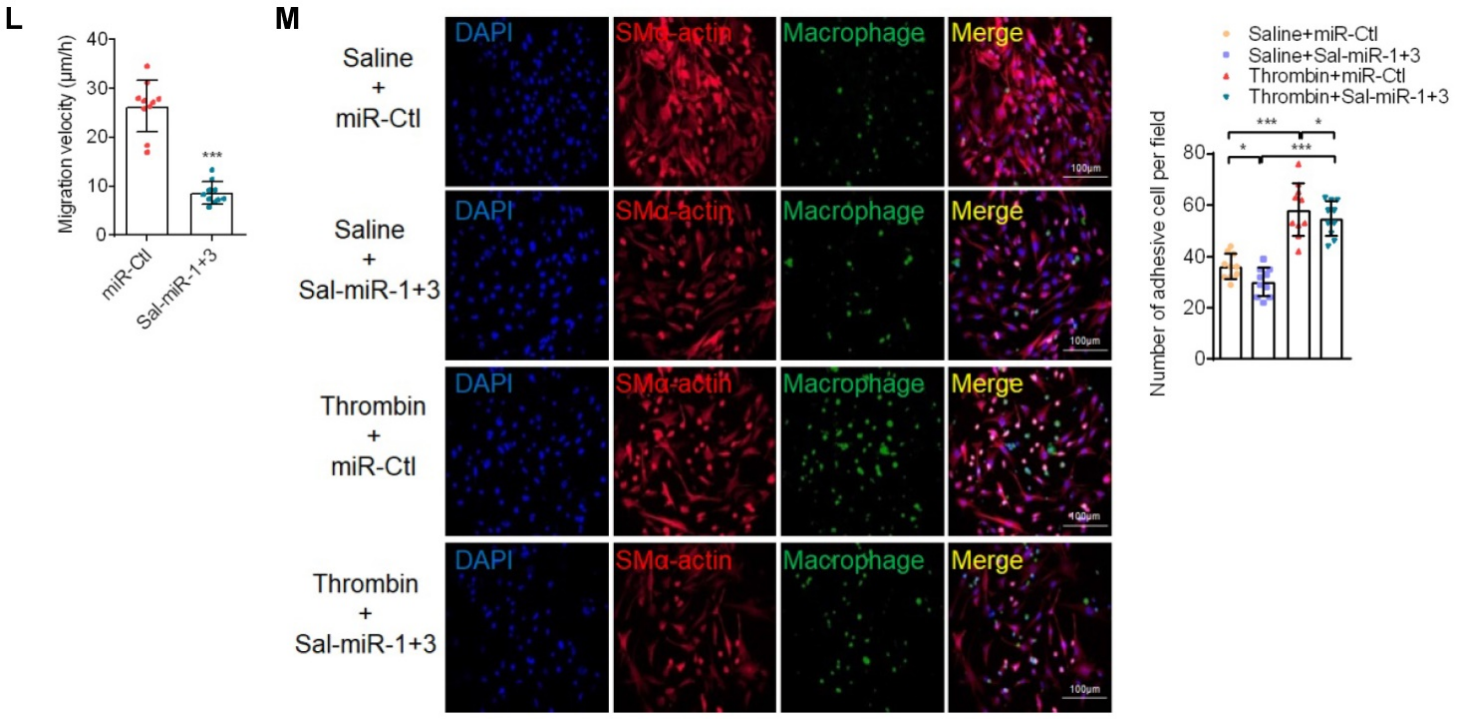
** Sal-miR-1 and 3 attenuate thrombin-induced migration of VSMCs and monocyte adhesion to VSMCs.** (**A and C**) Western blot analysis detected ICAM-1 and VCAM-1 expression in VSMCs treated with thrombin for the indicated doses and times. (**B and D**) Relative expression of ICAM-1 and VCAM-1 mRNA was examined by qRT-PCR. **P<0.01, ***P<0.001 versus 0 U/mL (B) or 0 h (**D**). (E) VSMCs were transfected with Sal-miR-1+3 or miR-Ctl, RNA was extracted and treated with sodium periodate. Relative level of Sal-miR-1 and 3 was detected by qRT-PCR. (**F-G**) VSMCs were transfected with Sal-miR-1+3 for 24 h and then treated or not with thrombin (1 U/mL) 24 h. The expression of ICAM-1 and VCAM-1 was analyzed by Western blotting (F) and qRT-PCR (G). (**H**) VSMCs were treated as in (F). The cells were fixed, stained for F-actin with TRITC-phalloidin and with DAPI to visualize nuclei, and imaged with a laser scanning confocal microscope. Scale bars=25 µm. (**I**) VSMCs were treated as in (F), a scratch wound assay was performed and photomicrographed. Relative migration rate was measured by Image J. n=10 fields, Scale bars=200 µm. (**J**) Boyden chamber assay performed with VSMCs treated as in (F). Migratory cell numbers per field were measured by Image J. n=10 fields, Scale bars=200 µm. (**K**) Thrombin-stimulated migration of VSMCs transfected with Sal-miR-1+3 was traced by using time-lapse imaging. (**L**) Migration distances of cells per hour were measured by Image J. n=10 cells. (**M**) Macrophage adhesion to VSMCs treated as in (F) was evaluated by staining macrophage marker with anti-MAC-2. Scale bars=200 µm. The numbers of macrophages adhered to VSMCs per field were measured by Image J. n=10 fields. Bar graphs show mean±SEM from 3 independent experiments (n=3). ND, Not Detected. Student's t-test, one-way ANOVA test or repeated Measures ANOVA: *P<0.05, **P<0.01, ***P<0.001 versus the corresponding control.

Next, we assessed the effects of Sal-miR-1 and 3 on ICAM-1 and VCAM-1 expression in cultured VSMCs. First, we used thrombin, which is one of the factors contributing to the development of intimal hyperplasia after arterial injury 27, to stimulate mouse VSMCs and showed that thrombin significantly upregulated the expression levels of VCAM-1and ICAM-1 mRNA and proteins in a dose- (Figure 2A-B) and time-dependent manner (Figure 2C-D). Then, VSMCs were transfected with Sal-miR-1 and 3, and these two miRNAs in VSMCs were confirmed be resistant to oxidation by sodium periodate (Figure 2E)*.* In the further experiments, VSMCs transfected with Sal-miR-1 and 3 were treated with thrombin, and Western blotting and qRT-PCR showed that Sal-miR-1 and 3 transfection largely abrogated the upregulation of VCAM-1 and ICAM-1 expression by thrombin both at the protein and mRNA levels (Figure 2F-G). We also used immunofluorescence staining to examine the expression of these two adhesion molecules in VSMCs transfected with Sal-miR-1 and 3 and then treated or not with thrombin. The results revealed that Sal-miR-1 and 3 transfection obviously reduced the expression of ICAM-1 and VCAM-1 regardless of treatment with thrombin (Figure S2A).

Considering that reorganization of the actin cytoskeleton is necessary for cell migration and adhesion 28, we examined the effects of thrombin and Sal-miR-1 and 3 on actin filaments. The results showed that thrombin treatment markedly reduced actin filaments, as evidenced by staining of TRITC-phalloidin, whereas transfecting VSMCs with Sal-miR-1 and 3 reduced depolymerization of filamentous actin (F-actin) induced by thrombin, with actin filaments being recruited into thick and long actin bundles (Figure 2H). Consistent with changes of the actin cytoskeleton, a scratch wound healing assay and transwell membrane assay showed that Sal-miR-1 and 3 strongly inhibited thrombin-induced migration of VSMCs compared with VSMCs transfected with miR-Ctl (Figure 2I-J). To further validate these observations, we used a time-lapse imaging experiment to measure the movement of VSMCs, where thrombin was added to one side of the culture dish to establish a spatial gradient. We showed that miR-Ctl-transfected VSMCs exhibited a marked time-dependent migration, but the movement of Sal-miR-1 and 3-transfected VSMCs was significantly decreased (Figure 2K-L). Accordingly, we used the cell co-culture system to examine bone marrow-derived macrophage or RAW264.7 macrophage adhesion to VSMCs, and found that thrombin promoted macrophage adhesion to VSMCs, whereas transfection of Sal-miR-1 and 3 counteracted the stimulatory effect of thrombin on cell adhesion (Figure 2M, Figure S2B). Meanwhile, mouse lymphocytes were also isolated from spleen and used in adhesion assays, and similar results were obtained (Figure S2C). Taken together, these data demonstrate that Sal-miR-1 and 3 attenuate the thrombin-induced VSMC migration and macrophage adhesion to VSMCs through suppressing adhesion molecule expression as well as by regulation of the actin cytoskeleton organization.

### Sal-miR-1 and 3 abrogate OTUD7B upregulation by thrombin via targeting the OTUD7B 3′UTR

**Figure 3:**
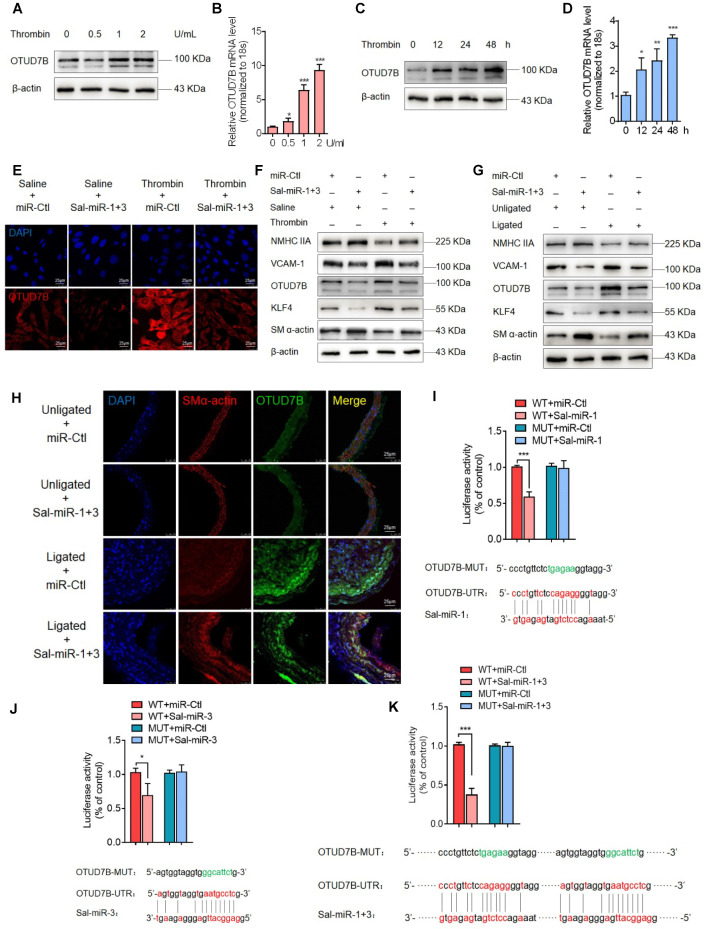

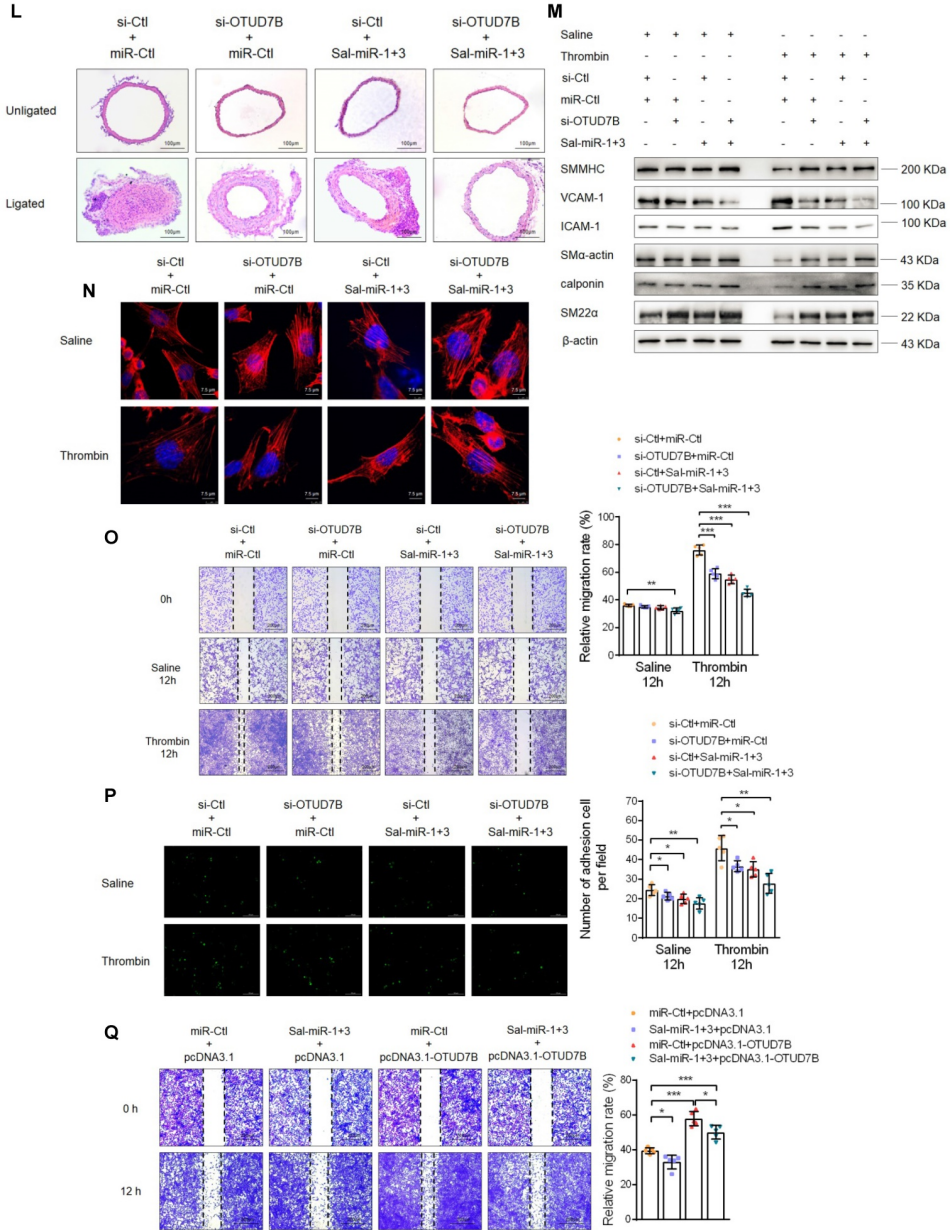
** Sal-miR-1 and 3 abrogate OTUD7B upregulation by thrombin via targeting the OTUD7B 3′UTR.** (**A-B**) VSMCs were treated with the different doses of thrombin for 24 h. OTUD7B expression was examined by Western blotting (A) and qRT-PCR (B). (**C-D**) VSMCs were treated with 1 U/mL thrombin for the indicated times. Western blotting (C) and qRT-PCR (D) detected OTUD7B expression. (**E**) Immunofluorescence staining detected OTUD7B expression in VSMCs transfected with Sal-miR-1+3 and then treated or not with 1 U/mL thrombin for 24 h. Red and blue staining indicates OTUD7B and the nuclei, respectively. Scale bar=25 µm. (**F**) VSMCs were transfected with Sal-miR-1+3 and then treated or not with 1 U/mL thrombin for 24 h. The expression of OTUD7B, KLF4, VCAM-1, SM α-actin, and NMHC IIA was determined by Western blotting. (**G**) The carotid arteries injured by ligation were *in situ*-transfected with Sal-miR-1+3. The expression of OTUD7B, KLF4, VCAM-1, SM α-actin, and NMHC IIA was determined by Western blotting. (**H**) Immunofluorescent staining for SM α-actin and OTUD7B was performed on sections from the carotid arteries transfected or not with Sal-miR-1+3. Red, green and blue staining indicates SM α-actin, OTUD7B and the nuclei, respectively. Scale bars=25 µm. (**I-K**) Luciferase reporter assays were performed in HEK293A cells transfected with OTUD7B 3'UTR reporter vector containing the wild-type or mutated Sal-miR-1, Sal-miR-3 or Sal-miR-1+3 binding site in the presence or absence of Sal-miR-1, Sal-miR-3 or Sal-miR-1+3. The pmirGLO vector was used as negative control. The Sal-miR-1 (I), Sal-miR-3 (J) and Sal-miR-1+3 (K) binding sites in the 3'UTR of the mouse OTUD7B mRNA are showed in red; the mutated sites are shown in green. (**L**) Representative hematoxylin and eosin staining of cross-sections from unligated and ligated carotid arteries transfected with miR-Ctl+si-Ctl (n=5), miR-Ctl+si-OTUD7B (n=5), si-Ctl+Sal-miR-1+3 (n=5), or Sal-miR-1+3+si-OTUD7B (n=5). Scale bars=100 µm. (**M**) VSMCs were transfected with si-OTUD7B, Sal-miR-1+3 or Sal-miR-1+3+si-OTUD7B and then treated or not with 1 U/mL thrombin for 24 h. The expression of SMMHC, ICAM-1, VCAM-1, SM α-actin, calponin and SM22α was determined by Western blotting. (**N**) VSMCs were treated as in (M). The cells were fixed, stained for F-actin with TRITC-phalloidin and with DAPI to visualize nuclei, and imaged with a laser scanning confocal microscope. Scale bars=7.5 µm. (**O**) VSMCs were treated as in (M), a scratch wound assay was performed and photomicrographed. Relative migration rate was measured by Image J. n=5 fields, Scale bars=200 µm. (**P**) Macrophage adhesion to VSMCs treated as in (M) was evaluated by staining macrophage marker with anti-MAC-2. Scale bars=10 µm. The numbers of macrophages adhered to VSMCs per field were measured by Image J. n=5 fields. (**Q**) VSMCs were transfected with Sal-miR-1+3 and/or pcDNA3.1-OTUD7B for 24 h. A scratch wound assay was performed and photomicrographed. Relative migration rate was measured by Image J. n=5 fields. All experiments were performed for three independent experiments. Student's t-test, one-way ANOVA test or repeated Measures ANOVA: *P<0.05, **P<0.01, ***P<0.001 versus the corresponding control.

Because plant-derived miRNAs are known to be capable of regulating mammalian gene expression by binding to the 3' UTR of target genes 19, 29, we utilized the bioinformatic databases miRanda (http://www.microrna.org/microrna/home.do), Targetscan (http://www.targetscan.org/vert_72/) and RNAhybrid (https://bibiserv.cebitec.uni-bielefeld.de/rnahybrid/) to predict Sal-miR-1 and 3 targets (Figure S3A, Table S1). We found that the deubiquitinase OTUD7B (also known as Cezanne) contains the putative Sal-miR-1 and 3-binding sites in its 3' UTR. We first observed that thrombin significantly increased OTUD7B expression both at the protein and mRNA levels in a dose- and time-dependent manner (Figure 3A-D). Next, we confirmed that Sal-miR-1 or Sal-miR-3 alone or together significantly inhibited the expression of OTUD7B mRNA and protein, but did not affect KLF3 and KLF10 expression, indicating the specificity of these two miRNA target recognition (Figure S3B-C). Importantly, when VSMCs were transfected with Sal-miR-1 and 3 and then treated with thrombin, the upregulation of OTUD7B by thrombin was greatly abolished (Figure 3E-F). Moreover, using a carotid artery ligation model, we showed that *in situ* transfection of the ligated carotid artery with Sal-miR-1 and 3 also markedly decreased the expression of OTUD7B and intimal hyperplasia induced by vascular injury compared with that transfected with miR-Ctl, as indicated by Western blot analysis and immunofluorescence staining (Figure 3G-H). Correspondingly, Sal-miR-1 and 3 also downregulated the level of KLF4 and VCAM-1 proteins regardless of thrombin treatment or artery injury, accompanied by a significant increase in SM α-actin and NMHC IIA expression compared with their corresponding control. To assess translational relevance of these observations, we also examined the effect of Sal-miR-1 and 3 on human VSMCs. The results showed that stimulation of human VSMCs with thrombin could reduce NMHC IIA expression and enhance the expression of OTUD7B and KLF4, which could be largely reversed by transfecting Sal-miR-1 and 3 (Figure S3D). To further confirm whether OTUD7B is a direct target of Sal-miR-1 and 3, we co-transfected cells with Sal-miR-1 or Sal-miR-3 alone or together, both of which bind to the different sites on OTUD7B 3′UTR, along with OTUD7B 3' UTR- or its mutant (OTUD7B-MUT)-mediated luciferase reporter. The results revealed that transfection of Sal-miR-1 or Sal-miR-3 alone or together significantly reduced the luciferase activity mediated by wild-type OTUD7B 3' UTR, but had no significant effect on the luciferase activity mediated by its mutant. The combination of these two miRNAs had a stronger inhibitory effect on luciferase activity than on of them alone (Figure 3I-K, Figure S3E)*.* Together, these data indicate that Sal-miR-1 and 3 negatively regulate OTUD7B expression via directly targeting the 3' UTR of OTUD7B mRNA.

Furthermore, we observed the effects of OTUD7B knockdown on the vascular protective effects of Sal-miR-1 and 3. The results showed that knockdown of OTUD7B further strengthened the vascular protective effect of Sal-miR-1 and 3, as evidenced by morphological analysis of intimal hyperplasia (Figure 3L). Also, Sal-miR-1 and 3-induced upregulation of SMC-specific contractile proteins (SM α-actin, SM22α, calponin and SMMHC), which were suppressed in thrombin-treated VSMCs, was largely enhanced by OTUD7B knockdown (Figure 3M). Moreover, OTUD7B inhibition also largely increased the inhibitory effects of Sal-miR-1 and 3 on thrombin-induced VSMC migration (Figure 3O) and adhesion (Figure 3P), accompanied by a corresponding change in actin cytoskeleton morphology (Figure 3N) and the expression of VCAM-1 and ICAM-1 (Figure 3M). Reversely, OTUD7B overexpression mediated by pcDNA3.1-OTUD7B partially blocked Sal-miR-1 and 3 inhibition of VSMC migration (Figure 3Q). These findings further suggest that OTUD7B is indispensable for the vascular protective effects of Sal-miR-1 and 3.

### OTUD7B downregulation by Sal-miR-1 and 3 attenuates KLF4 protein levels

**Figure 4:**
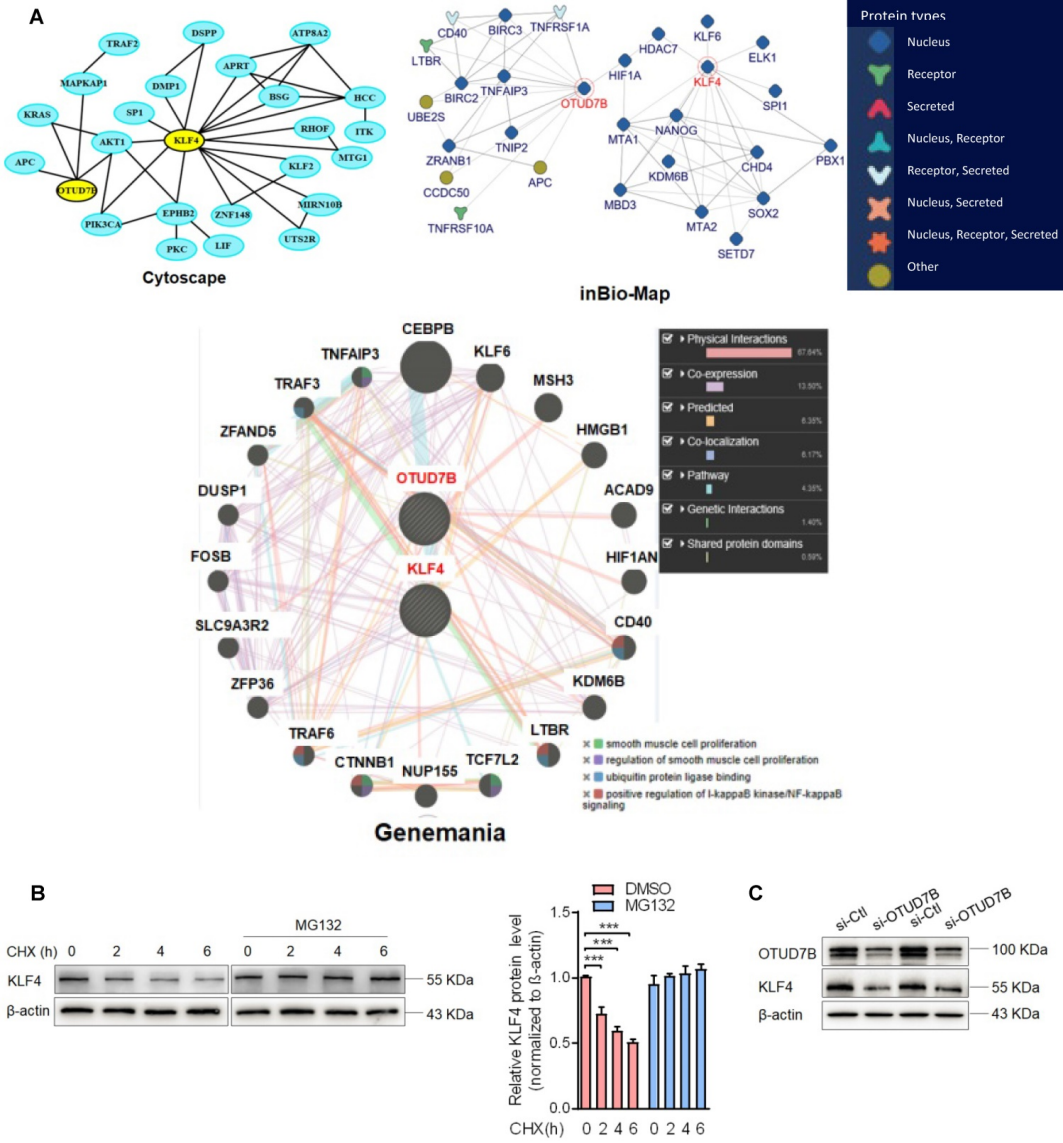

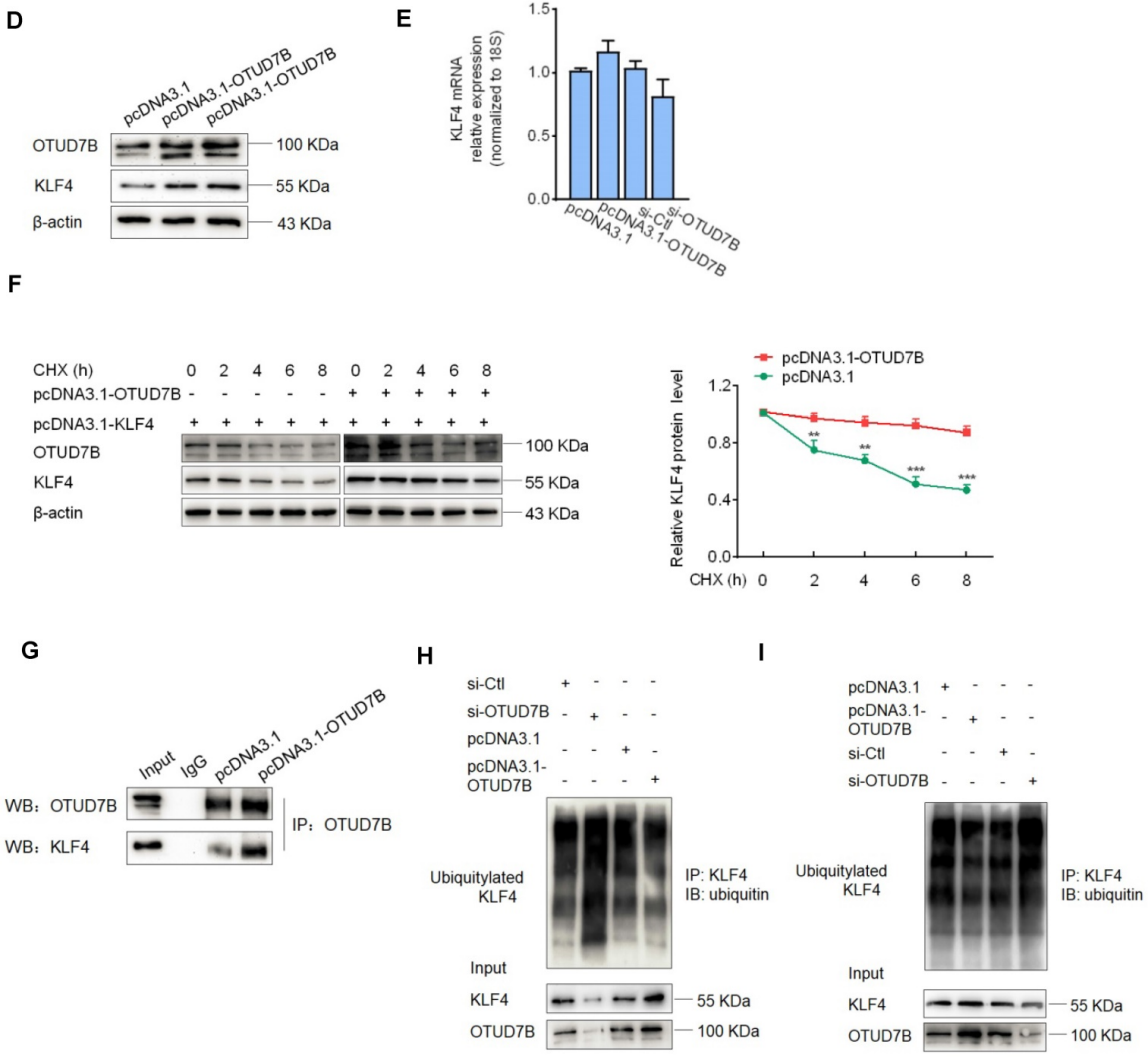
** OTUD7B downregulation by Sal-miR-1 and 3 attenuates KLF4 protein levels*.*** (**A**) Bioinformatics analysis of the proteins interacting with OTUD7B by three protein-protein interactions databases. Images were obtained from the Cytoscape, inBio-Map and Genemania online database (Cytoscape: http://apps.cytoscape.org/apps/agilentliteraturesearch; inBio-Map: https://www.intomics.com/inbio/map.html#search; Genemania: http://genemania.org). The potential proteins associated with OTUD7B were marked. (**B**) VSMCs were treated with 0.03 mg/mL cycloheximide (CHX), along with or without MG132 treatment. Cells were harvested at the indicated times, and KLF4 was detected by Western blotting, whereas band intensities that were measured are shown on the right. (**C**) VSMCs were transfected with si-Ctl or si-OTUD7B for 24 h, and the expression of OTUD7B and KLF4 was analyzed by Western blotting. (**D**) VSMCs were transfected with pcDNA3.1 or pcDNA3.1-OTUD7B for 24 h, and the expression of OTUD7B and KLF4 was analyzed by Western blotting. (**E**) VSMCs were transfected with pcDNA3.1-OTUD7B, si-OTUD7B or their corresponding control for 24 h. KLF4 expression was analyzed by qRT-PCR. (**F**) The Flag-tagged KLF4 expression vectors alone or together with GFP-tagged OTUD7B expression vectors were transiently transfected into HEK293A cells, after 24 h of transfection, 0.03 mg/mL CHX was added to each plate, and then incubated for 0, 2, 4, 6, and 8 h, respectively. Protein levels of KLF4 and OTUD7B were detected by Western blotting. (**G**) VSMCs were transfected with pcDNA3.1-OTUD7B or pcDNA3.1 for 24 h, and then anti-OTUD7B immunoprecipitates (IP) were analyzed by Western blot (WB) for OTUD7B and KLF4 co-sedimentation. IgG was used as negative control. (**H**) VSMCs were transfected with pcDNA3.1-OTUD7B, si-OTUD7B or their corresponding control for 24 h, and then treated with MG132 for 6 h. KLF4 was immunoprecipitated with anti-KLF4 antibody and immunoblotted with anti-ubiquitin antibody. (**I**) HEK293A cells were transfected for 24 h with pcDNA3.1-OTUD7B, si-OTUD7B or their corresponding control, and then treated with MG132 for 6 h. Cell lysates were incubated with anti-KLF4 antibody, and the immunoprecipitates were analyzed by immunoblotting with anti-ubiquitin antibody. All experiments were performed three independent experiments, and the results are presented as the mean ± SEM. Repeated Measures ANOVA or Student's t-test: **P<0.01, ***P<0.001 versus the corresponding control.

To clarify which substrate is regulated and deubiquitinated by OTUD7B in the context of VSMC migration and adhesion, we used 3 protein-protein interaction prediction databases (Cytoscape: http://apps.cytoscape.org/apps/agilentliteraturesearch; inBio-Map: https://www.intomics.com/inbio/map.html#search; Genemania: http://genemania.org) to search for putative proteins interacting with OTUD7B. As a result, KLF4 exists in the protein-protein interaction network for OTUD7B and KLF4 (Figure 4A). Because VSMC migration and adhesion are closely associated with VSMC phenotype switch regulated by KLF4, we investigated whether OTUD7B regulates KLF4 expression level at the post-translational level. We first used the cycloheximide (CHX) chase experiment to determine the stability of KLF4 protein. The results showed that CHX decreased the protein level of KLF4 in a time-dependent manner, and the half-life of KLF4 in VSMCs was found to be approximately 6 h. Addition of the proteasome inhibitor MG132 resulted in a marked increase in KLF4 protein stability (Figure 4B). Further, using loss- and gain-of-function experiments, we confirmed that knockdown of OTUD7B attenuated KLF4 protein levels, whereas the overexpression of OTUD7B had the opposite effect. However, OTUD7B knockdown or overexpression did not significantly influence KLF4 mRNA level (Figure 4C-E), indicating that OTUD7B regulates KLF4 protein levels through deubiquitinating KLF4, thereby inhibiting KLF4 proteolysis. Consistently, in HEK293 cells and VSMCs enforced to express OTUD7B and KLF4, OTUD7B overexpression obviously enhanced KLF4 protein stability (Figure 4F, Figure S4). In the further study, we utilized co-immunoprecipitation experiments to examine the interaction of OTUD7B with KLF4 and found that KLF4 was co-immunoprecipitated by anti-OTUD7B antibody. The specificity of their interaction was verified by increased association of OTUD7B with KLF4 in OTUD7B-overexpressed cells (Figure 4G). Also, we examined the effects of OTUD7B knockdown or overexpression on KLF4 ubiquitination in VSMCs. When endogenous OTUD7B was knocked down by si-OTUD7B, the levels of ubiquitylated KLF4 were substantially elevated (Figure 4H). Reversely, OTUD7B overexpression in 293A cells markedly reduced the accumulation of ubiquitylated KLF4, whereas its knockdown had an opposite effect (Figure 4I). These data indicated that OTUD7B interacts with and deubiquitylates KLF4 and OTUD7B downregulation by Sal-miR-1 and 3 attenuates KLF4 protein levels.

### KLF4 negatively regulates NMHC IIA expression, and downregulation of KLF4 by Sal-miR-1 and 3 reduces KLF4 repression of the expression of NMHC IIA and thus increases NMHC IIA expression level

**Figure 5:**
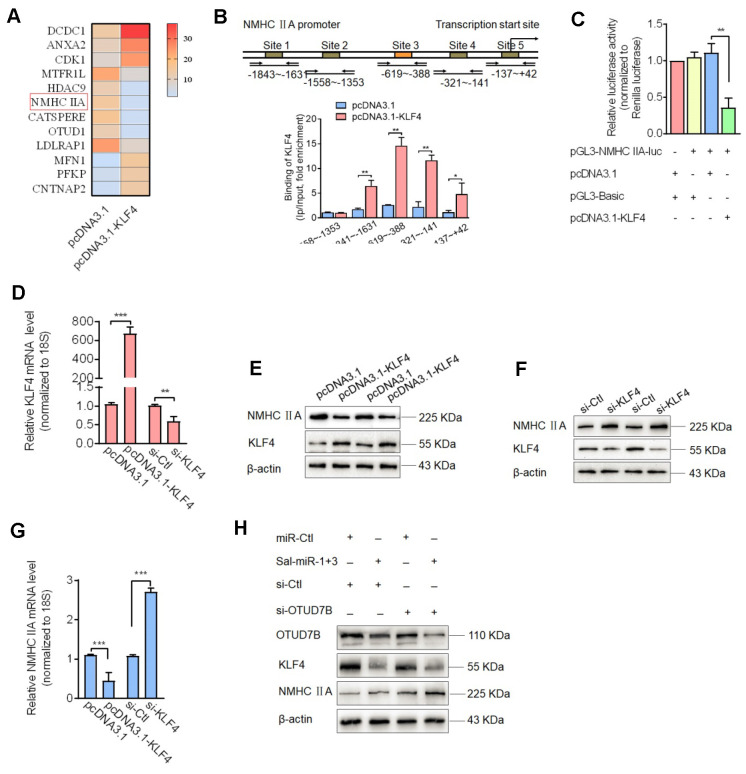
***D*ownregulation of KLF4 by Sal-miR-1 and 3 increases NMHC IIA expression level.** (**A**) A subset of the differentially expressed mRNAs detected in pcDNA3.1- or pcDNA3.1-KLF4-transfected VSMCs with microarray analysis was selected and summarized. (**B**) A schematic map of the -1843 to +42 bp region of the NMHC IIA promoter showing the positions of KLF4-binding sites. VSMCs were transfected with pcDNA3.1 or pcDNA3.1-KLF4 for 24 h, and then formaldehyde-cross-linked chromatin was extracted and immunoprecipitated with anti-KLF4 antibody. Nonimmune IgG was used as negative control for immunoprecipitation. Immunoprecipitated DNA containing KLF4 binding sites was amplified by PCR using mouse NMHC IIA promoter-specific primers. (**C**) VSMCs were co-transfected with KLF4 expression plasmids and NMHC IIA promoter-reporter plasmids for 24 h. Luciferase activity was measured using the dual luciferase reporter assay system. (**D-G**) VSMCs were transfected with pcDNA3.1-KLF4, si-KLF4 or their corresponding control for 24 h. The expression of KLF4 and NMHC IIA was analyzed by qRT-PCR (D-G) and Western blot (**E-F**). (**H**) VSMCs were transfected with Sal-miR-1+3 and/or si-OTUD7B for 24 h. The expression of OTUD7B, KLF4 and NMHC IIA was analyzed by Western blotting. All results are presented as the mean±SEM from 3 independent experiments (n=3). Student's t-test: *P<0.05, **P<0.01, ***P<0.001 versus the corresponding control.

Because Sal-miR-1 and 3 downregulated KLF4 protein levels by attenuating OTUD7B expression, we sought to determine KLF4 target genes associated with induction of VSMC migration. To determine KLF4 target genes associated with induction of VSMC migration, we performed RNA microarray analysis of KLF4-overexpressing VSMCs vs. control cells (Table S2) and identified 12 significantly differentially expressed genes, of which 6 were downregulated and 6 upregulated (using a more than 2-fold change as the criteria for differential expression) (Fig. 5A). Among the genes downregulated by KLF4 overexpression, NMHC IIA is a well-known cytoskeletal protein 30 and closely correlated with cell adhesion and migration 8, 13. Thus, we focused on the KLF4 regulation of the expression of NMHC IIA as well as the role of NMHC IIA in VSMC migration and monocyte adhesion to VSMCs. Next, chromatin immunoprecipitation (ChIP) with KLF4 antibodies followed by PCR demonstrated that KLF4 directly bound to upstream sequences of the NMHC IIA promoter harboring the -1841 to -141 bp, and the overexpression of KLF4 resulted in a 14-fold increase in KLF4 occupancy of the region between -619 and -388 bp (Figure 5B). Indeed, when searching KLF4 binding sites on the promoter region of NMHC IIA in transcription factor prediction database, we found that there exist putative KLF4 binding sites in the proximal promoter region of NMHC IIA (Figure 5B). To provide further evidence that KLF4 regulates the expression of NMHC IIA, we used a dual-luciferase reporter assay to determine the effect of KLF4 overexpression on the activity of the NMHC IIA promoter. The results revealed that co-transfection of KLF4 expression vector with NMHC IIA-directed luciferase reporter significantly reduced luciferase activity, with the activity being reduced to 30% of the control (Figure 5C). Consistent with this result, KLF4 overexpression decreased, whereas KLF4 knockdown increased NMHC IIA expression both at the protein and mRNA levels (Figure 5D-G). However, when the expression of KLF4 in VSMCs was downregulated by transfecting cells with Sal-miR-1 and 3 as well as by knocking down the expression of OTUD7B with small interfering RNA against OTUD7B (si-OTUD7B), NMHC IIA expression was largely increased (Figure 5H). Together, these findings indicated that Sal-miR1+3 enhance the expression levels of NMHC IIA gene by suppressing OTUD7B expression and thus reducing KLF4 protein levels.

### Sal-miR-1 and 3 upregulate NMHC IIA expression, and increased NMHC IIA represses VSMC migration and monocyte adhesion to VSMCs via maintaining the contractile phenotype of VSMCs

**Figure 6:**
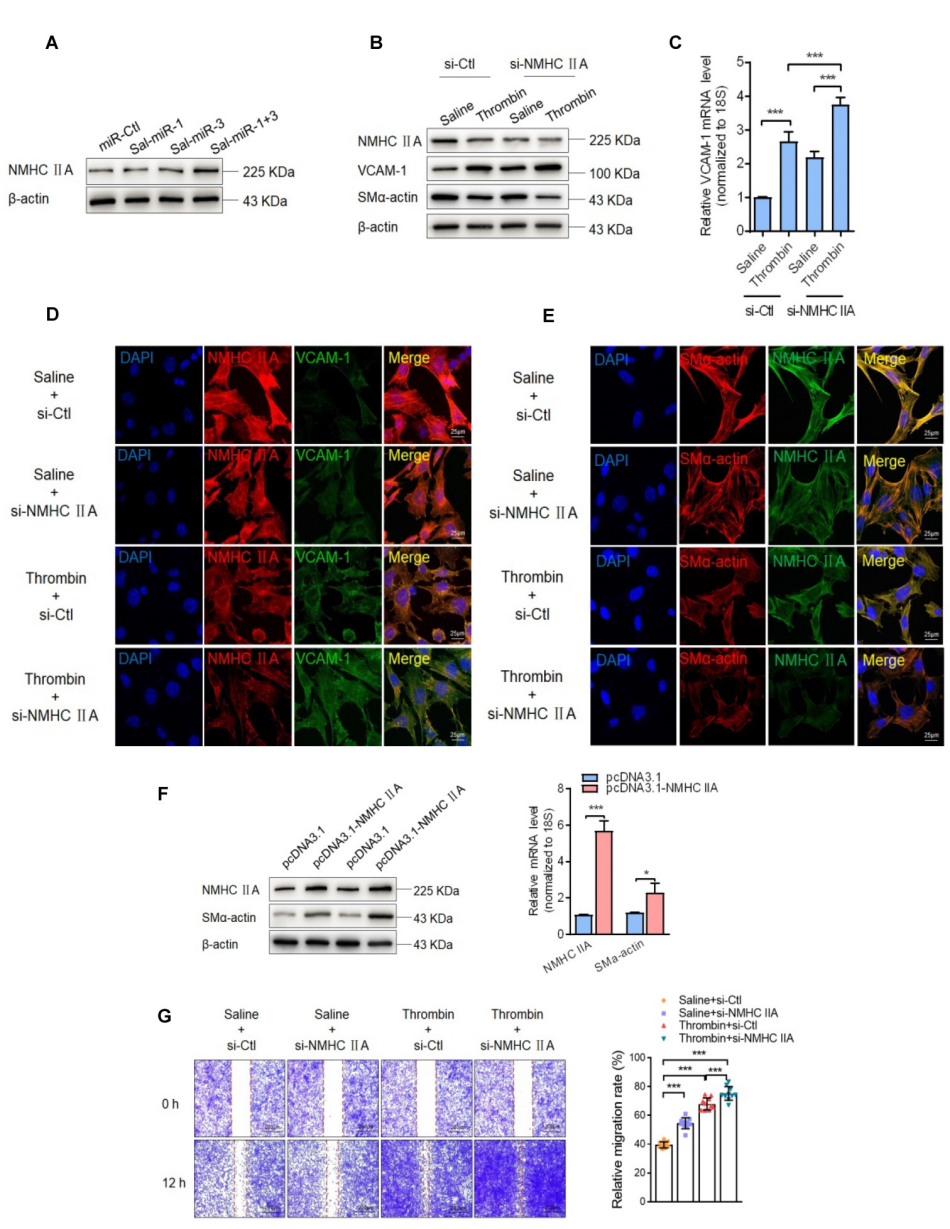

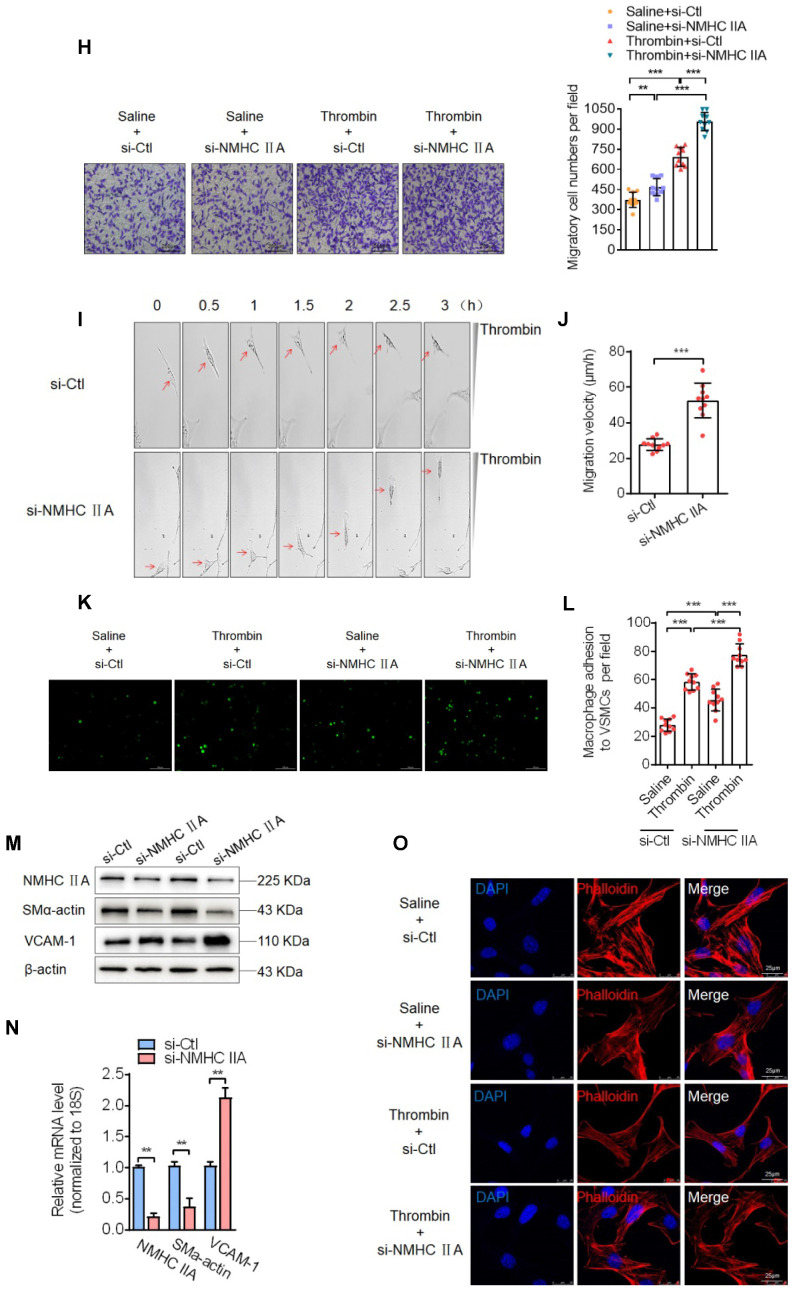

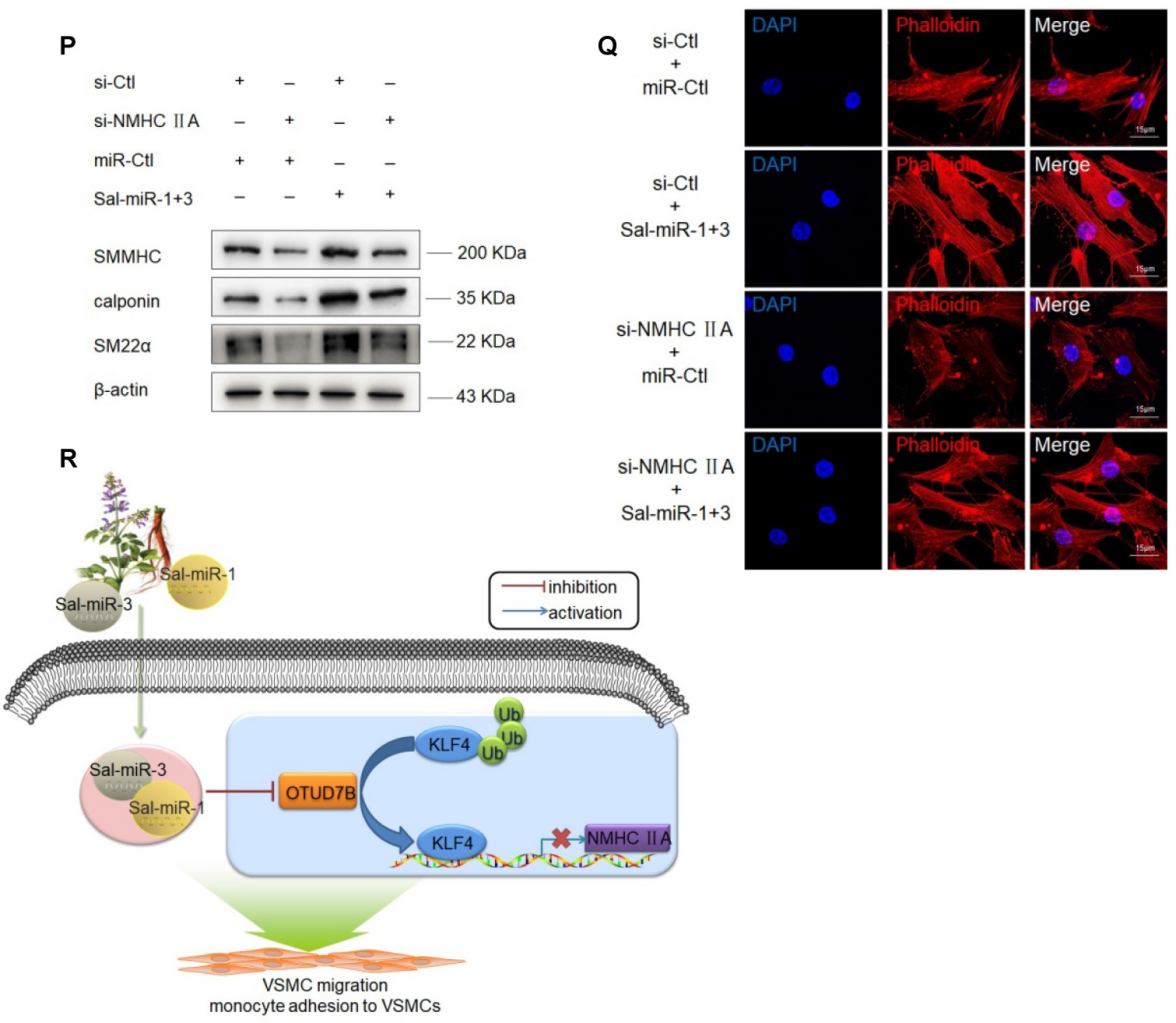
**Sal-miR-1 and 3 upregulate NMHC IIA expression levels, and increased NMHC IIA represses VSMC migration and monocyte adhesion to VSMCs***.* (**A**) VSMCs were transfected with miR-Ctl, Sal-miR-1, Sal-miR-3 or Sal-miR-1 and 3 for 24 h. NMHC IIA expression was analyzed by Western blotting. (**B-C**) VSMCs were transfected with si-Ctl or si-NMHC IIA for 24 h and then treated or not with thrombin (1 U/mL) for 24 h. The expression of NMHC IIA, VCAM-1 and SM α-actin was analyzed by Western blotting (B) and qRT-PCR (C). (**D**) VSMCs were treated as in (B). NMHC IIA, VCAM-1 and their co-localization were examined by immunofluorescence staining. Red, green and blue staining indicates NMHC IIA, VCAM-1 and the nuclei, respectively. Yellow staining indicates co-localization of NMHC IIA with VCAM-1. Scale bars=25 µm. (**E**) VSMCs were treated as in (B). Immunofluorescence staining for SM α-actin (red), NMHC IIA (green) and the nuclei (blue). Scale bars=25 µm. (**F**) VSMCs were transfected with pcDNA3.1 or pcDNA3.1-NMHC IIA for 24 h. The expression of NMHC IIA and SM α-actin was analyzed by Western blotting and qRT-PCR. (**G**) VSMCs were transfected with si-Ctl or si-NMHC IIA for 24 h and then treated or not with thrombin (1 U/mL). A scratch wound assay was performed and photomicrographed. Relative migration rate was measured by Image J. n=10 fields. (**H**) Boyden chamber assay performed with VSMCs treated as in (G). Migratory cell numbers per field were measured by Image J. n=10 fields. Scale bars=200 µm. (**I-J**) Thrombin-stimulated migration of si-NMHC IIA-transfected VSMCs was traced by using time-lapse imaging. Migration distances were measured by Image J. n=10 cells. (**K-L**) VSMCs were treated as in (G). RAW264.7 cell adhesion to VSMCs was observed by staining macrophage marker with anti-MAC-2. Scale bars=100 µm. Data are presented as the mean ± SD from three independent experiments, n=10 fields. (**M-N**) VSMCs were transfected with si-Ctl or si-NMHC IIA for 24 h. The expression of NMHC IIA, VCAM-1 and SM α-actin was analyzed by Western blotting (M) and qRT-PCR (N). (**O**) VSMCs were treated as in (G). The cells were fixed, stained fluorescently for F-actin with TRITC-phalloidin, and imaged with a laser scanning confocal microscope. Scale bars=25 µm. (**P**) VSMCs were transfected with Sal-miR-1+3 and/or si-NMHC IIA for 24 h. The expression of SMMHC, calponin and SM22α was analyzed by Western blotting. (**Q**) VSMCs were treated as in (P). The cells were fixed, stained fluorescently for F-actin with TRITC-phalloidin, and imaged with a laser scanning confocal microscope. Scale bars=15 µm. (**R**) Proposed model for Sal-miR-1 and 3-regulated OTUD7B/KLF4/NMHC IIA axis. Bar graphs show mean±SEM from 3 independent experiments (n=3). Student's t-test or one-way ANOVA test: *P<0.05, **P<0.01, ***P<0.001 versus the corresponding control.

It has been well known that the contractile phenotype of VSMCs is maintained by activating a subset of SRF-dependent genes encoding contractile and cytoskeletal proteins, and that KLF4 behaves as a repressor of VSMC differentiation, inhibiting these gene expression 31-33. The above findings suggested that Sal-miR-1 and 3-regulated OTUD7B/KLF4/NMHC IIA pathway modulates VSMC migration and adhesion, which are fundamental characteristics of the proliferative phenotype. Next, we investigated the mechanisms underlying NMHC IIA-mediated VSMC migration and adhesion. First, we showed that NMHC IIA expression was substantially elevated in Sal-miR-1 and 3-transfected VSMCs (Figure 6A). Further, we knocked down the endogenous expression of NMHC IIA with si-NMHC IIA and observed the effects of thrombin on the expression of VCAM-1 and VSMC marker gene SM α-actin. The results of Western blotting, qRT-PCR and immunofluorescence staining revealed that thrombin markedly reduced NMHC IIA and SM α-actin expression, accompanied by a dramatic increase in VCAM-1 expression levels, and NMHC IIA knockdown further strengthened the impact of thrombin on VCAM-1 and SM α-actin expression (Figure 6B-E). In contrast, overexpression of NMHC IIA in VSMCs could significantly enhance the expression of SM α-actin (Figure 6F). Besides SM α-actin, SM22α, SMMHC and calponin expression was also examined as a measure of VSMC phenotype. Overexpression of NMHC IIA obviously upregulated the expression of SM22α, SMMHC and calponin genes (Figure S5A). Moreover, a scratch wound healing assay and Boyden chamber transwell assay showed that thrombin treatment obviously promoted VSMC migration compared with that treated with vehicle, and NMHC IIA knockdown further enhanced the stimulatory effect of thrombin on VSMC migration (Figure 6G-H). To validate these observations, time-lapse imaging experiments were also performed, and get the same results (Figure 6I-J). In the further experiments, we examined that effect of thrombin alone or together with NMHC IIA knockdown on macrophage adhesion to VSMCs by using a VSMC-macrophage co-culture system***.***The results showed that thrombin promoted macrophage adhesion to VSMCs, and transfection of si-NMHC IIA further enhanced the adhesion between these two cells induced by thrombin (Figure 6K-L).

Because the migration and adhesion of WSMCs are closely related to VSMC phenotype switching and are regulated by the changes in the actin cytoskeleton 34, we further investigated the effect of NMHC IIA knockdown on the phenotype and cytoskeleton morphology of VSMCs. Knockdown of endogenous NMHC IIA by si-NMHC IIA markedly decreased the expression levels of SM α-actin, a marker of the differentiated state of VSMCs*,* both at the protein and mRNA levels, with a concomitant increase in the expression of VCAM-1 gene (Figure 6M-N). Similarly, besides SM α-actin, knockdown of NMHC IIA also reduced the expression of SM22α, SMMHC, and calponin genes (Figure S5B). Correspondingly, phalloidin staining also showed that knockdown of NMHC IIA substantially attenuated actin stress fiber formation, consistent with the changes of expression of SM α-actin (Figure 6O). Furthermore, we further examined whether NMHC IIA knockdown could block Sal-miR-1 and 3-mediated regulation of VSMC phenotype. The results showed that NMHC IIA knockdown by si-NMHC IIA could partially block Sal-miR-1 and 3-mediated actin stress fiber formation and upregulated SM22α, SMMHC and calponin expression (Figure 6P-Q). Taken together, these data indicate that NMHC IIA modulates SM α-actin expression and thus affects the organization of the actin cytoskeleton by some unrecognized mechanisms*,* which in turn maintains the contractile phenotype of VSMCs, decreasing VSMC migration and monocyte adhesion to VSMCs.

## Discussion

Previous studies have shown that, in addition to playing an important role in coagulation, thrombin, which is released from platelets in response to vascular injury, also stimulates the inflammatory response 35 and enhances migration and abnormal proliferation of VSMCs, which has the central place in vascular remodeling 36. Mechanistic investigation revealed that thrombin activates protein kinase C (PKC) via the interaction with the G protein coupled receptor (GPCR), leading to the activation of mitogen-activated protein kinase (MAPK) signaling partway 36. Thrombin also induces the expression of nuclear proteins that constitute the transcription factor AP-1, which participates in transactivation of several early growth response genes implicated in VSMC proliferation 35. Moreover, thrombin induces F-actin remodeling through activating RhoA signaling in endothelial cells 37. Despite advances in the role of thrombin in vascular remodeling, the precise mechanisms underlying thrombin-induced VSMC migration and adhesion remain largely unclear. Therefore, elucidating the molecular mechanism of vascular remodeling induced by thrombin and investigating how to block thrombin-mediated induction of vascular remodeling have important significance for preventing and treating vascular remodeling.

This study offers many new findings: 1) First, we established, for the first time, *Salvia miltiorrhiza*-derived microRNAs, namely, Sal-miR-1 and Sal-miR-3, as the novel bioactive components that possess cardiovascular protective actions, and that they can regulate the expression of mouse and human OTUD7B gene via targeting the OTUD7B 3′UTR in a cross-kingdom manner. 2) Second, although OTUD7B, as a pivotal negative regulator of the non-canonical NF-κB pathway, is known to be important for regulating B-cell responses 38, 39 and T-cell homeostasis 40, little is known about the role of OTUB7B in the regulation of VSMC function. We show here that OTUB7B upregulates the level of KLF4 protein, a stem cell pluripotency protein 41, through interacting with and deubiquitylating KLF4, a repressor of VSMC differentiation, whereas OTUD7B downregulation by Sal-miR-1 and 3 attenuates KLF4 protein level. 3) Third, we show for the first time that KLF4 downregulation elicited by Sal-miR-1 and 3-reduced expression of OTUD7B relieves KLF4 repression of the expression of VSMC differentiation genes (e.g. SM α-actin, SM22α, calponin and SMMHC) and thus increases their expression levels. 4) We documented that increased expression of VSMC differentiation genes represses VSMC migration and macrophage adhesion to VSMCs via maintaining the differentiation phenotype of VSMCs. 5) Finally, our findings add important knowledge to the bioactive components of *Salvia miltiorrhiza*. Sal-miR-1 and 3-regulated OTUD7B/KLF4/NMHC IIA axis may represent a therapeutic target for vascular remodeling (Figure 6R).

In this study, our results showed that Sal-miR-1 and 3 reduced the upregulation of the OTUD7B mRNA and protein induced by thrombin. Results of luciferase reporter assays using OTUD7B 3′UTR or its mutant plasmids demonstrated that Sal-miR-1 and 3 could target the 3'UTR of OTUD7B mRNA and then inhibited OTUD7B expression. OTUD7B is a member of the A20 family of deubiquitinating enzymes 42. Previous studies have shown that OTUD7B inhibited NF-κB pathway by deconjugating K63-polyubiquitin chains from RIP-1 and TRAF6, suggesting that it may have roles in inhibition of cancer progression 42, 43. Indeed, Wang et al. found that OTUD7B reduction is correlated with poor prognosis and thus may serve as a prognostic factor for hepatocellular carcinoma patients 44. In vascular endothelial cells, OTUD7B could reduce inflammation and injury in response to ischemia-reperfusion 45. Additionally, Pareja et al. reported that OTUD7B was overexpressed in a large percentage of breast cancers and that high expression levels of OTUD7B correlated with poor patient prognosis 46. However, the role of OTUD7B in VSMCs has not been clarified. In this study, we showed that OTUD7B expression was markedly upregulated in thrombin-stimulated VSMCs, accompanied by an increased VCAM-1 expression and migration of VSMCs, consistent with the previous observation that thrombin is a potent modulator of various pro-inflammatory markers in atherosclerotic lesions 36. Sal-miR-1 and Sal-miR-1 obviously decreased the expression of OTUD7B and intimal hyperplasia induced by vascular injury, suggesting that OTUD7B plays the pro-inflammatory and pro-proliferation effects for VSMCs in response to vascular injury. Our findings and previous observations indicate that OTUD7B may be a pleiotropic molecule that possesses divergent functions, implying that the biological function of OTUD7B is context-dependent.

VSMC contractile gene expression and, thus differentiation, is under direct transcriptional control by SRF and cofactors, such as myocardin and myocardin-related transcription factors (MRTFs), which act as powerful transcriptional coactivators of SRF in mammalian cells. On the other hand, KLF4 functions as a repressor of VSMC differentiation, inhibiting the expression of SMC differentiation markers such as SM α-actin, SM22α, and SMMHC 31-33. Previous studies have indicated that the underlying mechanisms of the downregulation of VSMC differentiation markers (e.g. SM α-actin, SM22α, and SMMHC) by KLF4 in VSMCs include: (1) direct KLF4 binding to the TGF-β control element within the promoter/enhancer regions of SMC differentiation genes; (2) interaction with SRF; (3) repression of expression of an SRF coactivator, myocardin; (4) recruitment of HDAC2 to SMC genes, resulting in deacetylation of histone H4, chromatin compaction, and loss of myocardin/SRF binding; and (5) suppression of DNA methylation 47-49. VSMC migration and adhesion are closely associated with VSMC phenotype switching regulated by KLF4 47. KLF4 drives phenotypic switching by the suppression of VSMC marker genes 31, 50, 51. Considering the crucial role of KLF4 in VSMC phenotypic switching as well as that KLF4 protein level is regulated by ubiquitination 52, 53, we hypothesized that OTUD7B could regulate KLF4 protein stability through deubiquitylating KLF4. As anticipated, co-immunoprecipitation experiments revealed that OTUD7B interacted with and deubiquitylated KLF4. Correspondingly, OTUD7B knockdown decreased, whereas its overexpression increased, KLF4 protein levels. To date, little is known about the role of deubiquitinases (DUBs) in the regulation of VSMC phenotypes. In endothelial cells, OTUD7B is a key regulator of inflammatory responses to ischemia, and this process is controlled by a balance between ubiquitination and deubiquitination. OTUD7B reduces inflammation and injury in response to ischemia-reperfusion by suppressing tumor necrosis factor receptor-associated factor 6 (TRAF6) polyubiquitination 48. Considering that KLF4 is a transcription factor required for VSMC phenotype switching, and its stability is regulated by ubiquitination-mediated protein degradation, it is reasoned that OTUD7B downregulation induced by Sal-miR-1 and 3 resulted in a decrease in KLF4 protein levels.

Non-muscle myosin II (NM II), an actin-binding protein, is central in the control of cell adhesion, cell migration and tissue architecture 8. NMHC IIA is an important component of non-muscle myosin II and has been known to be a known effector of Rho-ROCK signaling pathway and frequently to be upregulated in gastric cancer 54, 55. A recent study showed that NMHC IIA expression is regulated by miR-647/SRF regulatory pathway; that is, miR-647 directly binds to the 3' UTR of SRF mRNA, and SRF activates NMHC IIA gene transcription by binding to the CArG box located at the NMHC IIA promoter 30. However, the actual relationship between KLF4 and NMHC IIA expression remains to be clarified. It has been known that NM II molecules assemble into bipolar filaments, and the latter links actin filaments together in thick bundles that form cellular structures such as stress fibres 8. Thus, NM II can use its actin cross-linking and contractile functions to regulate the actin cytoskeleton. Moreover, NM IIA-mediated contraction, calpain-dependent cleavage of adhesion components and microtubule targeting coordinately induce adhesion disassembly. Additionally, NM II also is a central integrator of external force during cell and tissue differentiation 8. Here, we found that there exist KLF4 binding sites in the proximal promoter region of NMHC IIA gene and confirmed that KLF4 directly bound to its binding sites and suppressed NMHC IIA gene transcription. Remarkably, Sal-miR-1 and 3 increased the expression levels of NMHC IIA by inhibiting OTUD7B expression and thus reducing KLF4 protein levels. Further, increased NMHC IIA maintained the contractile phenotype of VSMCs through modulating the organization of the actin cytoskeleton, thereby attenuating VSMC migration and monocyte adhesion to VSMCs.

Our findings reveal, for the first time, that OTUD7B upregulates the level of KLF4 protein through interacting with and deubiquitylating KLF4. Importantly, OTUD7B downregulation by Sal-miR-1 and 3 results in a reduction in KLF4 protein level, which in turn relieves KLF4 repression of the expression of SM α-actin, SM22α, and SMMHC, thus facilitating VSMC contractile gene (VSMC differentiation gene) expression. Based on these evidences, it is reasonable to conclude that downregulation of KLF4 protein level induced by Sal-miR-1 and 3/OTUD7B contributes to increased expression of VSMC contractile genes, while knockdown or overexpression of NMHC IIA in VSMCs affects SM α-actin expression through an as yet unidentified mechanism. Therefore, NMHC IIA regulation of VSMC contractile gene expression might be secondary or indirect. Our ongoing studies are designed to clarify these questions in more detail.

Taken together, our studies not only found the novel bioactive components from *Salvia miltiorrhiza* but also clarified the molecular mechanism underlying the inhibition of the migration of VSMCs and monocyte adhesion to VSMCs. These results add important knowledge to the pharmacological actions and bioactive components of *Salvia miltiorrhiza.* Sal-miR-1 and 3-regulated OTUD7B/KLF4/NMHC IIA axis may represent a therapeutic target for vascular remodeling.

## Supplementary Material

Supplementary figures and tables.

## Figures and Tables

**Table 1:** Primers for Real-time PCR and RT-PCR

Name	Sequences 5' to 3'
hbr-miR156	Forward: GCGTTGACAGAAGATAGAGAGC
	Reverse: TATGGTTGTTCACGACTCCTTCAC
ppt-miR414	Forward: CATCCTCATCATCCTCGTCC
	Reverse: TATGGTTGTTCACGACTCCTTCAC
gma-miR172b-5p	Forward: ACGGTAGCATCATCAAGATTCAC
	Reverse: TATGGTTGTTCACGACTCCTTCAC
Sal-miR-1	Forward: CGTAAAGACCTCTGATGAGAGTG
	Reverse: TATGGTTGTTCACGACTCCTTCAC
Sal-miR-3	Forward: GAGGCATTGAGGGAGAAGT
	Reverse: TATGGTTGTTCACGACTCCTTCAC
U6	Forward: CGCTTCGGCAGCACATATAC
	Reverse: TTCACGAATTTGCGTGTCATC
OTUD7B	Forward: TGGCAGCAAACACAGCAGAA
	Reverse: CTCCGTTGGAACCCAGATGC
TXA2R	Forward: GCTGCTCATCTACCTGCGTGTG
	Reverse: GCCTGGAGCTGTGAACTGAACC
ICAM-1	Forward: CTCATCCTGCGCTGTCTGGT
	Reverse: CCGGAGCTGCCTGACCTCGG
VCAM-1	Forward: CGTACCTGCTCAAGTGATGG
	Reverse: GTGTCTCCCTCTTTGACGCT
KLF3	Forward: AGGCCTCACTCACGGGATAC
	Reverse: AGAGAGGAAGGAGAACCGCC
KLF10	Forward: GCACAGTGTCCGATGGTGATGAG
	Reverse: ACGAAGGAGCTGGCTGAGACC
NMHC IIA	Forward: ACCGACTTCACCAGAGGCATCC
	Reverse: GCACCAGCCAGCGGAACATC
KLF4	Forward: CTAACCGTTGGCGTGAGGAACTC
	Reverse: TCTAGGTCCAGGAGGTCGTTGAAC
SM22α	Forward: CTAACCGTTGGCGTGAGGAACTC
	Reverse: TCTAGGTCCAGGAGGTCGTTGAAC
SMα-actin	Forward: CTAACCGTTGGCGTGAGGAACTC
	Reverse: TCTAGGTCCAGGAGGTCGTTGAAC
Calponin	Forward: CAAAGTCAATGTGGGAGTCAAG
	Reverse: CAGTTTGGGATCATAGAGGTGA
SMMHC	Forward: AAGCAGCTAAAGGACAAAACAG
	Reverse: ATGTCACATTGTCATTTAGCGG

**Table 2:** Primer sequences for ChIP

Name	Sequences 5' to 3'
site-1: NMHC IIA promoter-5	F:CACTGTAGCTGTCTTCACACACTCCA
	R: AAGCACGCCTGTCCTTGGC
site-2: NMHC IIA promoter-4	F: AGTCTTGAAC TCCCCCTCCTACC
	R:AAACTTACATTCTGAAGCCAAAAAGCAGCT
site-3: NMHC IIA promoter-3	F:AGAGGTACTAGCCAGAGCAAAGGT
	R: GAGGCTCTGACACTCCGCC
site-4: NMHC IIA promoter-2	F: AGCCCAGCGC GCCTGAGC
	R: CGGAGTGCTGAGATTCATCCTCC
site-5: NMHC IIA promoter-1	F:CCATCTCTTGTCTGTTTATAGGCAAAATCTG
	R: AGCTCTGGTGCGCGCCGC

## Abbreviations

VSMCs**: vascular smooth muscle cells
Sal: *Salvia miltiorrhiza*
qRT-PCR: quantitative RT-PCR
chr: chromosome
siRNA: short interfering RNA
miRNA: microRNA
EGFR: epidermal growth factor receptor
F-actin: filamentous actin
NMHC IIA: non-muscle myosin heavy chain IIA
WT: wild-type
I/M ratio: ratio of intimal/medial area
CHX: cycloheximide
ChIP: chromatin immunoprecipitation
SM α-actin: smooth muscle alpha actin
ICAM-1: intercellular adhesion molecule-1
VCAM-1: vascular cell adhesion molecule-1
OTUD7B: ovarian tumor deubiquitinase 7B
KLF4: krüppel-like factor 4
SM22α: smooth muscle protein 22α
PDGF-BB: platelet-derived growth factor-BB
TGF-β: transforming growth factor-β
Ang II: angiotensin II
