# Supplementary material for: Salvia miltiorrhiza-derived miRNAs suppress vascular remodeling through regulating OTUD7B/KLF4/NMHC IIA axis
Source: Theranostics. 2020 Jun 19;10(17):7787–811. doi: 10.7150/thno.46911 (PMC7359079; doi:10.7150/thno.46911)
Supplement: Supplementary file 1 — Supplementary figures and tables. [file thnov10p7787s1.pdf]

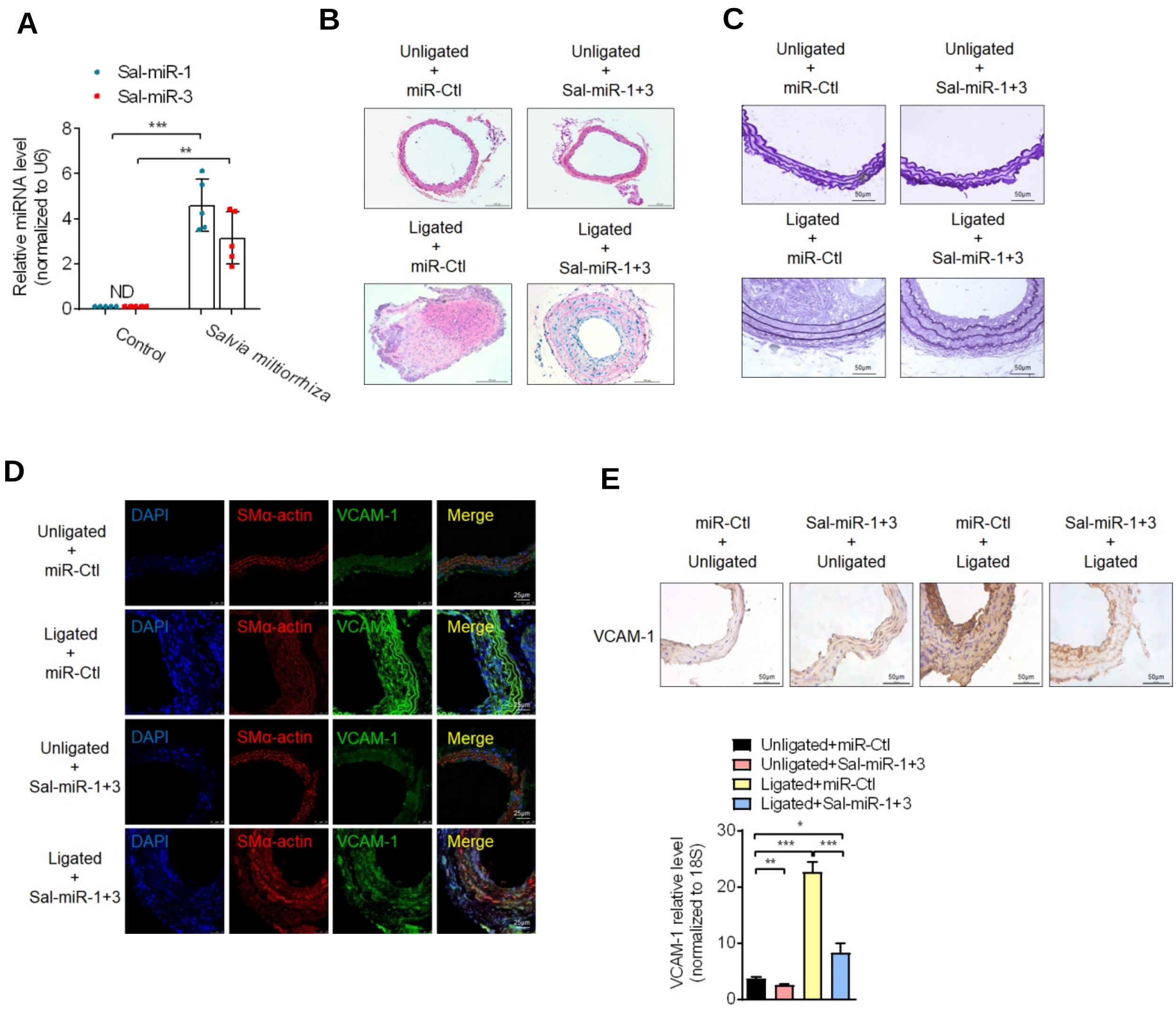

Supplemental Figure S1. (A) Levels of Sal-miR-1 and Sal-miR-3 in the serum of patients with atherosclerosis were determined by the qRT-PCR (n=5). (B) Representative hematoxylin and eosin staining of cross-sections from uninjured and injured carotid arteries of mice by intragastric administration of miR-Ctl (n=9) or Sal-miR-1+3 (n=9). Scale bars=100 μm. (C) Representative Elastic-van Gieson staining of cross-sections from uninjured and injured carotid arteries of mice by intragastric administration of miR-Ctl (n=9) or Sal-miR-1+3 (n=9). Scale bars=50 μm. (D) The unligated and ligated carotid arteries were transfected or not with Sal-miR-1+3. Immunofluorescence staining for SM α-actin (red), VCAM-1 (green) and the nuclei (blue) was performed. Scale bars=25 μm. (E) Immunohistochemical staining for VCAM-1 on cross-sections from the unligated and ligated carotid arteries transfected with miR-Ctl (n=9) or Sal-miR-1+3 (n=7). Quantitative analysis of VCAM-1-positive staining was performed by Image J. Bar graphs show mean±SEM from 3 independent experiments (n=3). Student's t-test or one-way ANOVA: **P*<0.05, ***P*<0.01, ****P*<0.001 versus the corresponding control.

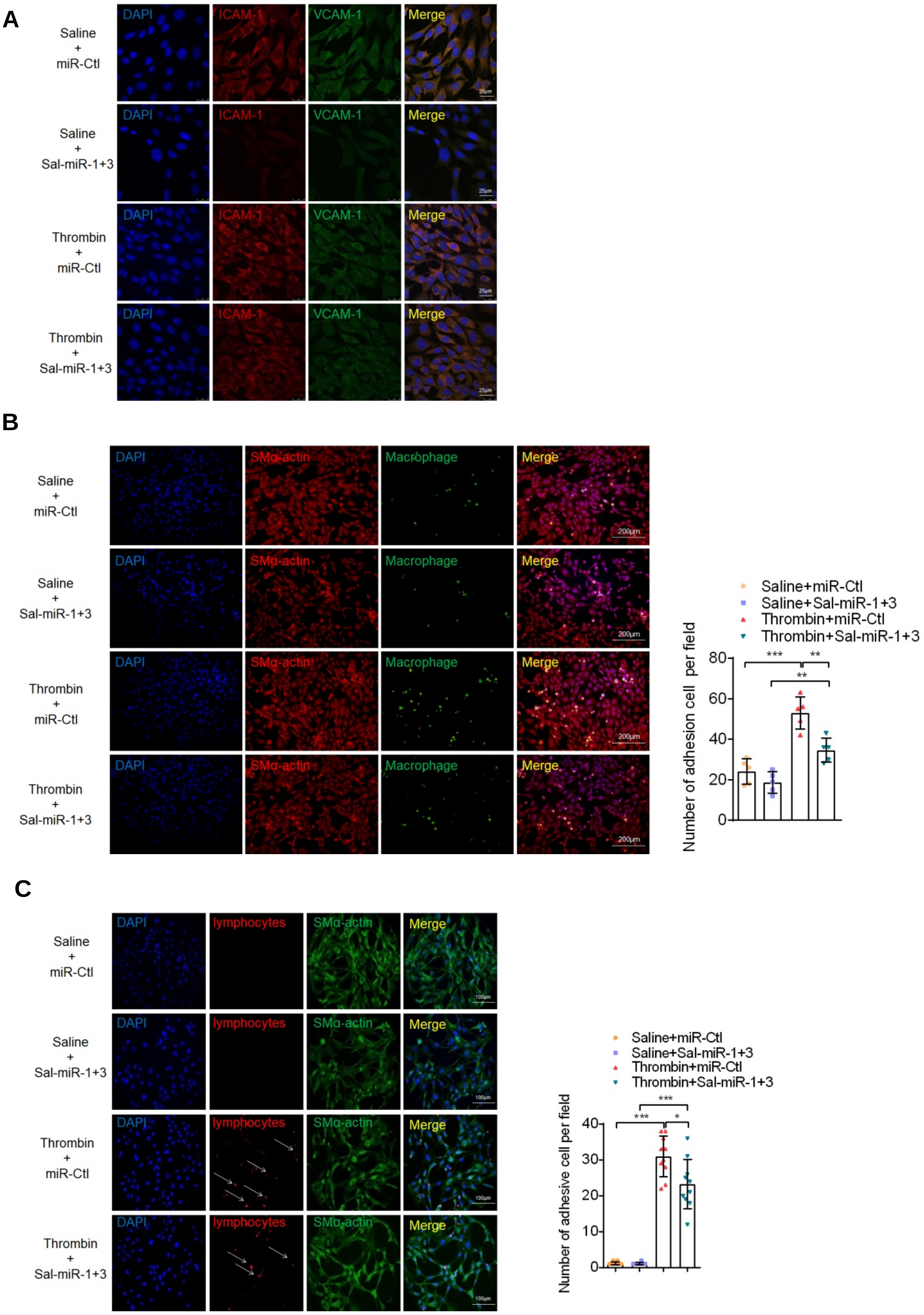
A
Saline + miR-Ctl
Saline + Sal-miR-1+3
Thrombin + miR-Ctl
Thrombin + Sal-miR-1+3
DAPI
ICAM-1
VCAM-1
Merge
25μm
B
Saline + miR-Ctl
Saline + Sal-miR-1+3
Thrombin + miR-Ctl
Thrombin + Sal-miR-1+3
DAPI
SMα-actin
Macrophage
Merge
200μm
Saline+miR-Ctl
Saline+Sal-miR-1+3
Thrombin+miR-Ctl
Thrombin+Sal-miR-1+3
Number of adhesion cell per field
0
20
40
60
80
***
**
**
C
Saline + miR-Ctl
Saline + Sal-miR-1+3
Thrombin + miR-Ctl
Thrombin + Sal-miR-1+3
DAPI
lymphocytes
SMα-actin
Merge
100μm
Saline+miR-Ctl
Saline+Sal-miR-1+3
Thrombin+miR-Ctl
Thrombin+Sal-miR-1+3
Number of adhesive cell per field
0
10
20
30
40
***
***
*

Supplemental Figure S2. (A) VSMCs were transfected with miR-Ctl or Sal-miR-1+3 and then treated or not with thrombin (1 U/mL) for 24 h, and then immunofluorescent staining for ICAM-1 and VCAM-1 was performed. Red, green and blue staining indicates ICAM-1, VCAM-1 and the nuclei, respectively. Scale bars=25 μm. (B) Bone marrow-derived macrophages adhesion to VSMCs treated as in (A) was evaluated by staining macrophage marker with anti-MAC-2. Scale bars=200 μm. The numbers of macrophages adhered to VSMCs per field were measured by Image J. n=5 fields. (C) VSMCs were treated as in (A), the lymphocyte adhesion to VSMCs was examined by staining with PKH26. Scale bars= 100 μm. The numbers of lymphocytes adhered to VSMCs per field were measured by Image J. n=9 fields. Data represent mean±SEM. Student's t-test or one-way ANOVA: *P<0.05, **P<0.01, ***P<0.001 versus the corresponding control.

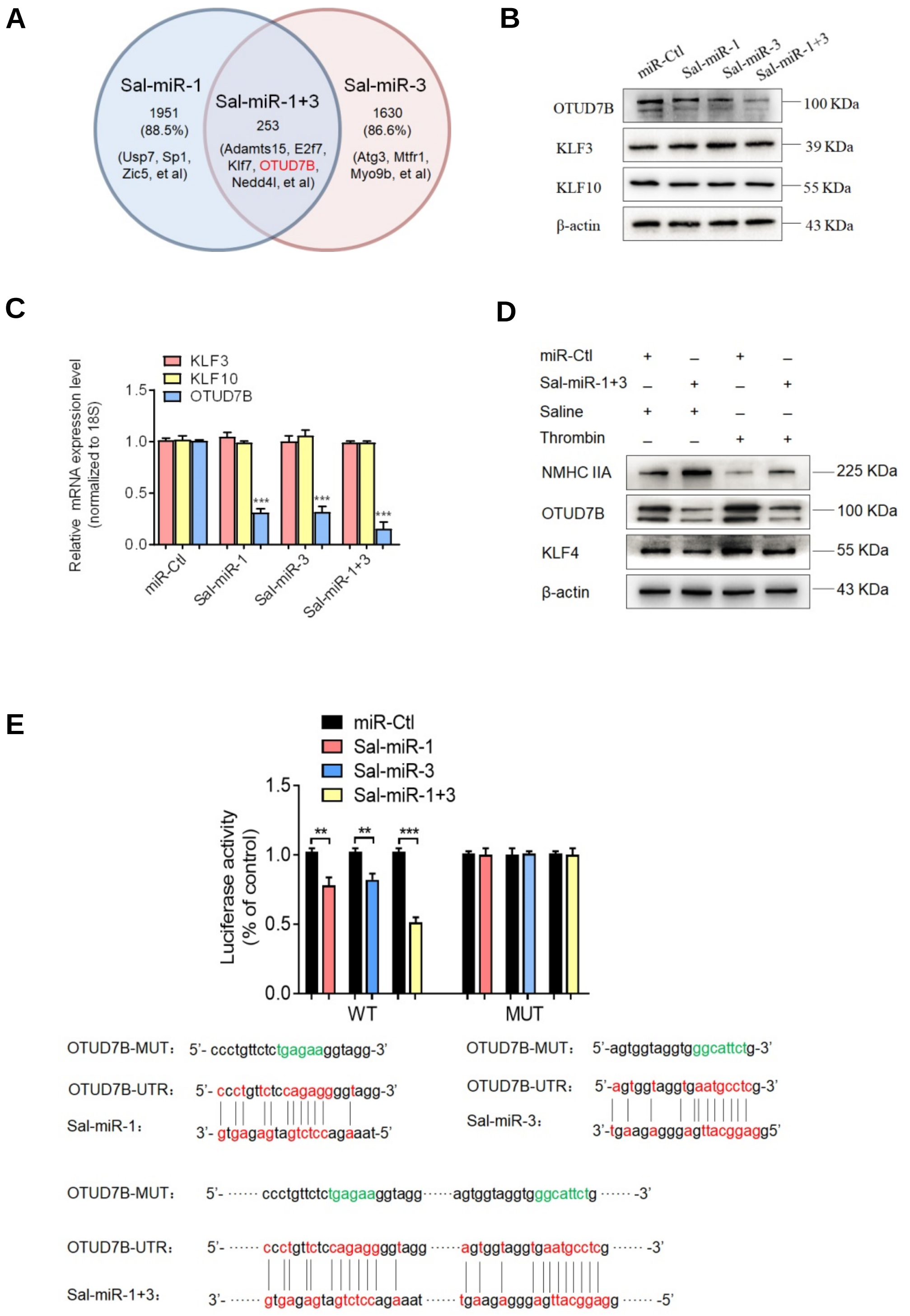
A
Sal-miR-1
1951
(88.5%)
(Usp7, Sp1, Zic5, et al)
Sal-miR-1+3
253
(Adamts15, E2f7, Klf7, OTUD7B, Nedd4l, et al)
Sal-miR-3
1630
(86.6%)
(Atg3, Mtfr1, Myo9b, et al)
B
miR-Ctl
Sal-miR-1
Sal-miR-3
Sal-miR-1+3
OTUD7B
KLF3
KLF10
β-actin
100 KDa
39 KDa
55 KDa
43 KDa
C
KLF3
KLF10
OTUD7B
Relative mRNA expression level (normalized to 18S)
1.5
1.0
0.5
0.0
***
miR-Ctl
Sal-miR-1
Sal-miR-3
Sal-miR-1+3
D
miR-Ctl
Sal-miR-1+3
Saline
Thrombin
NMHC IIA
OTUD7B
KLF4
β-actin
225 KDa
100 KDa
55 KDa
43 KDa
E
miR-Ctl
Sal-miR-1
Sal-miR-3
Sal-miR-1+3
Luciferase activity (% of control)
**
***
WT
MUT
OTUD7B-MUT: 5'- ccctgttctctgagaaggtagg-3'
OTUD7B-UTR: 5'- ccctgttctccagagggtagg-3'
Sal-miR-1: 3'- gtgagagtagtctccagaaat-5'
OTUD7B-MUT: 5'-agtggtaggtgggcattctg-3'
OTUD7B-UTR: 5'-agtggtaggtgaatgcctcg-3'
Sal-miR-3: 3'-tgaagagggagttacggagg5'
OTUD7B-MUT: 5'- ······ ccctgttctctgagaaggtagg······agtggtaggtgggcattctg ······ -3'
OTUD7B-UTR: 5'- ······ ccctgttctccagaggggtagg ······agtggtaggtgaatgcctcg ······ -3'
Sal-miR-1+3: 3'- ······ gtgagagtagtctccagaaat ······ tgaagagggagttacggagg ······ -5'

Supplemental Figure S3. (A) Bioinformatics prediction of potential Sal-miR-1 and Sal-miR-3 targets by 3 common miRNA databases. miRanda (http://www.microrna.org/microrna/home.do), Targetscan (http://www.targetscan.org/vert_72/) and RNAhybrid (https://bibiserv.cebitec.uni-bielefeld.de/rnahybrid/). (B-C) VSMCs were transfected with miR-Ctl, Sal-miR-1, Sal-miR-3, or Sal-miR-1+3 for 24 h. The expression of OTUD7B, KLF3 and KLF10 was determined by Western blotting (B) and qRT-PCR (C). (D) Human VSMCs were transfected with Sal-miR-1+3 and then treated or not with 1 U/mL thrombin for 24 h. The expression of OTUD7B, KLF4 and NMHC IIA was determined by Western blotting. (E) Luciferase reporter assays were performed in VSMCs transfected with OTUD7B 3'UTR reporter vector containing the wild-type or mutated Sal-miR-1, Sal-miR-3 or Sal-miR-1+3 binding site in the presence or absence of Sal-miR-1, Sal-miR-3 or Sal-miR-1+3. The pmirGLO vector was used as negative control. The Sal-miR-1, Sal-miR-3 and Sal-miR-1+3 binding sites in the 3'UTR of the mouse OTUD7B mRNA are showed in red; the mutated sites are shown in green. Bar graphs show mean±SEM from 3 independent experiments (n=3). Student's t-test or one-way ANOVA: **P<0.01, ***P<0.001 versus the corresponding control.

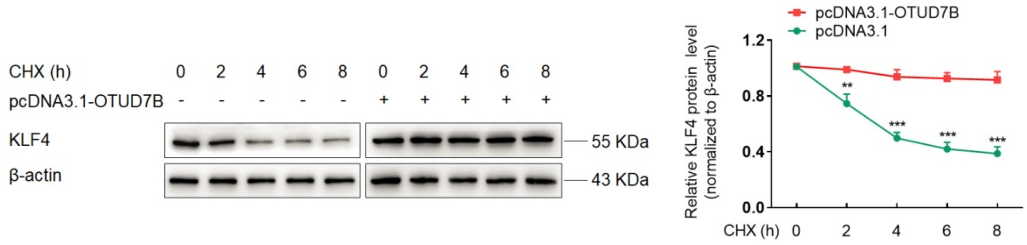

Supplemental Figure S4. The control vectors or OTUD7B expression vectors were transiently transfected into VSMCs, after 24 h of transfection, 30 μg/mL CHX was added to each plate, and then incubated for 0, 2, 4, 6, and 8 h, respectively. Protein levels of KLF4 were detected by Western blotting, whereas band intensities that were measured are shown on the right. Data represent mean±SEM from 3 independent experiments (n=3). Repeated Measures ANOVA or Student's t-test: ***P*<0.01, ****P*<0.001 versus the corresponding control.

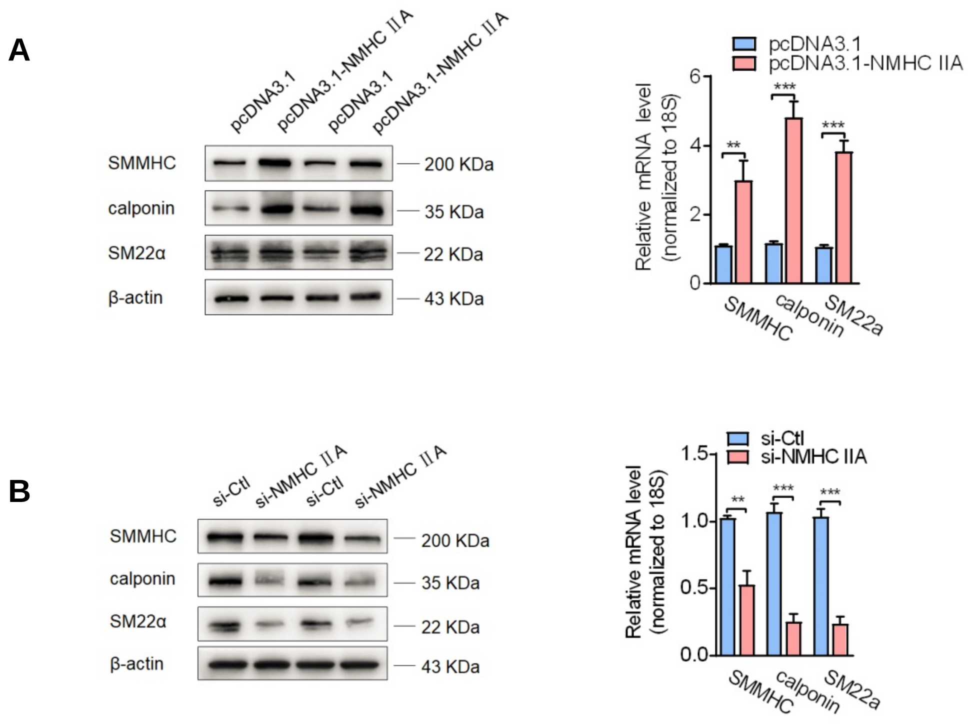

Supplemental Figure S5. (A) VSMCs were transfected with pcDNA3.1 or pcDNA3.1-NMHC IIA for 24 h. The expression of SMMHC, calponin and SM22α was analyzed by Western blotting and qRT-PCR. (B) VSMCs were transfected with si-Ctl or si-NMHC IIA for 24 h. The expression of SMMHC, calponin and SM22α was analyzed by Western blotting and qRT-PCR. Bar graphs show mean±SEM from 3 independent experiments (n=3). Student's t-test: ***P*<0.01, ****P*<0.001 versus the corresponding control.

Table S1. The common target genes of Sal-miR-1 and 3						
	ID	Gene	ID	Gene	ID	Gene
Sal-miR-1+3 targets	ENSMUSG00000039100	6-Mar	ENSMUSG00000006134	Crkl	ENSMUSG00000038143	Stox2
	ENSMUSG00000058706	0610030E20Rik	ENSMUSG00000026011	Ctla4	ENSMUSG00000024077	Strn
	ENSMUSG00000090066	1110002E22Rik	ENSMUSG00000041134	Cyyr1	ENSMUSG00000032437	Stt3b
	ENSMUSG00000054676	1600014C10Rik	ENSMUSG00000026883	Dab2ip	ENSMUSG00000020546	Stxbp4
	ENSMUSG00000036046	5031439G07Rik	ENSMUSG00000000346	Dazap2	ENSMUSG00000038045	Sult6b1
	ENSMUSG00000073386	9830107B12Rik	ENSMUSG00000041966	Dcaf17	ENSMUSG00000043866	Taf10
	ENSMUSG00000028127	Abcd3	ENSMUSG00000046818	Ddit4l	ENSMUSG00000029192	Tbc1d14
	ENSMUSG00000026348	Acmsd	ENSMUSG00000032097	Ddx6	ENSMUSG00000036667	Tcaf1
	ENSMUSG00000033453	Adamts15	ENSMUSG00000003166	Dgcr2	ENSMUSG00000028619	Tceanc2
	ENSMUSG00000029778	Adcyap1r1	ENSMUSG00000062393	Dgkk	ENSMUSG00000021275	Tecpr2
	ENSMUSG00000037605	Adgrl3	ENSMUSG00000021707	Dhfr	ENSMUSG00000032625	Thsd7a
	ENSMUSG00000028842	Ago3	ENSMUSG00000044716	Dok7	ENSMUSG00000020694	Tlk2
	ENSMUSG00000001211	Agpat3	ENSMUSG00000034973	Dopey1	ENSMUSG00000024736	Tmem132a
	ENSMUSG00000029772	Ahcyl2	ENSMUSG00000068536	Doxl2	ENSMUSG00000055296	Tmem245
	ENSMUSG00000024480	Ap3s1	ENSMUSG00000043671	Dpy19l3	ENSMUSG00000024614	Tmx3
	ENSMUSG00000019979	Apaf1	ENSMUSG00000020185	E2f7	ENSMUSG00000027692	Tnik
	ENSMUSG00000037509	Arhgef4	ENSMUSG00000035064	Eef2k	ENSMUSG00000020422	Tns3
	ENSMUSG00000026663	Atf6	ENSMUSG00000027293	Ehd4	ENSMUSG00000026848	Tor1b
	ENSMUSG00000028710	Atpaf1	ENSMUSG00000020091	Eif4ebp2	ENSMUSG00000032366	Tpm1
	ENSMUSG00000020564	Atxn7l1	ENSMUSG00000028546	Elavl4	ENSMUSG00000043909	Trp53bp1
	ENSMUSG00	Avpr2	ENSMUSG00	Elf2	ENSMUSG00	Ttll12

	000031390		000037174		000016757	
	ENSMUSG00000074892	B3galt5	ENSMUSG00000031103	Elf4	ENSMUSG00000051747	Ttn
	ENSMUSG00000040270	Bach2	ENSMUSG00000022995	Enah	ENSMUSG00000031723	Txnl4b
	ENSMUSG00000035021	Baz1a	ENSMUSG00000019768	Esr1	ENSMUSG00000043621	Ubxn10
	ENSMUSG00000078786	BC024978	ENSMUSG00000030275	Etnk1	ENSMUSG00000057948	Unc13d
	ENSMUSG00000022247	Brix1	ENSMUSG00000044465	Fam160a2	ENSMUSG00000026696	Vamp4
	ENSMUSG00000046449	C77370	ENSMUSG00000060568	Fam78b	ENSMUSG00000054455	Vapb
	ENSMUSG00000033417	Cacul1	ENSMUSG00000028218	Fam92a	ENSMUSG00000021614	Vcan
	ENSMUSG00000043541	Casc1	ENSMUSG00000030691	Fchsd2	ENSMUSG00000018377	Vezf1
	ENSMUSG00000038127	Ccdc50	ENSMUSG00000043683	Fem1a	ENSMUSG00000066735	Vkorc1l1
	ENSMUSG00000079084	Ccdc82	ENSMUSG00000033386	Frrs1	ENSMUSG00000028753	Vwa5b1
	ENSMUSG00000016493	Cd46	ENSMUSG00000055026	Gabrg3	ENSMUSG00000021266	Wars
	ENSMUSG00000038147	Cd84	ENSMUSG00000052557	Gan	ENSMUSG00000041245	Wnk3
	ENSMUSG00000024780	Cdc37l1	ENSMUSG00000026893	Gca	ENSMUSG00000030093	Wnt7a
	ENSMUSG00000026361	Cdc73	ENSMUSG00000041440	Gk5	ENSMUSG00000022100	Xpo7
	ENSMUSG00000006191	Cdkal1	ENSMUSG00000074219	Gm10644	ENSMUSG00000047694	Yipf6
	ENSMUSG00000052353	Cemip	ENSMUSG00000078117	Gm16485	ENSMUSG00000041995	Zbed3
	ENSMUSG00000008206	Cers4	ENSMUSG00000093752	Gm20716	ENSMUSG00000022708	Zbtb20
	ENSMUSG00000034203	Chchd4	ENSMUSG00000094081	Gm20826	ENSMUSG00000037553	Zdhhc18
	ENSMUSG00000014668	Chfr	ENSMUSG00000094484	Gm21244	ENSMUSG00000025786	Zdhhc3
	ENSMUSG00000030077	Chl1	ENSMUSG00000092225	Gm2381	ENSMUSG00000053985	Zfp14
	ENSMUSG00000014077	Chp1	ENSMUSG00000033852	Gm28042	ENSMUSG00000057101	Zfp180

	ENSMUSG00000060002	Chpt1	ENSMUSG00000098374	Gm28043	ENSMUSG00000038535	Zfp280d
	ENSMUSG00000029516	Cit	ENSMUSG00000107705	Gm45062	ENSMUSG00000046556	Zfp319
	ENSMUSG00000004317	Clcn5	ENSMUSG00000073427	Gm4924	ENSMUSG00000099689	Zfp383
	ENSMUSG00000026317	Cln8	ENSMUSG00000096742	Gm6367	ENSMUSG00000028358	Zfp618
	ENSMUSG00000031789	Cngb1	ENSMUSG00000026754	Golga1	ENSMUSG00000046311	Zfp62
	ENSMUSG00000004665	Cnn2	ENSMUSG00000031545	Gpat4	ENSMUSG00000022987	Zfp641
	ENSMUSG00000056941	Commd7	ENSMUSG00000028096	Gpr89	ENSMUSG00000058093	Zfp729b
	ENSMUSG00000026489	Coq8a	ENSMUSG00000041078	Grid1	ENSMUSG00000063383	Zfp947
	ENSMUSG00000031450	Grk1	ENSMUSG00000046881	Olfr374	ENSMUSG00000070605	Zfp992
	ENSMUSG00000021730	Hcn1	ENSMUSG00000070382	Olfr391-ps	ENSMUSG00000096433	Zfp994
	ENSMUSG00000020721	Helz	ENSMUSG00000073974	Olfr551	ENSMUSG00000061894	Zscan20
	ENSMUSG00000007617	Homer1	ENSMUSG00000073896	Olfr716	ENSMUSG00000030346	Rad51ap1
	ENSMUSG00000037234	Hook3	ENSMUSG00000091873	Olfr732	ENSMUSG00000051615	Rap2a
	ENSMUSG00000045777	Ifitm10	ENSMUSG00000095917	Olfr740	ENSMUSG00000038555	Reep2
	ENSMUSG00000000159	Igsf5	ENSMUSG00000048391	Olfr843	ENSMUSG00000042671	Rgs8
	ENSMUSG00000005364	Il5ra	ENSMUSG00000047050	Olfr914	ENSMUSG00000039917	Rhbdd2
	ENSMUSG00000040329	Il7	ENSMUSG00000038495	Otud7b	ENSMUSG00000028098	Rnf115
	ENSMUSG00000003500	Impdh1	ENSMUSG00000028736	Pax7	ENSMUSG00000038876	Rnf146
	ENSMUSG00000040865	Ino80d	ENSMUSG00000020553	Pctp	ENSMUSG00000025203	Scd2
	ENSMUSG00000041879	Ipo9	ENSMUSG00000064330	Pde6h	ENSMUSG00000026589	Sec16b
	ENSMUSG00000058975	Kcnc1	ENSMUSG00000026773	Pfkfb3	ENSMUSG00000050010	Shisa3
	ENSMUSG00000062785	Kcnc3	ENSMUSG00000020359	Phykpl	ENSMUSG00000022372	Sla

	**ENSMUSG00000051726**	**Kcnf1**	**ENSMUSG00000050229**	**Pigm**	**ENSMUSG00000015314**	**Slamf6**
	**ENSMUSG00000030180**	**Kdm5a**	**ENSMUSG00000032462**	**Pik3cb**	**ENSMUSG00000089774**	**Slc5a3**
	**ENSMUSG00000020653**	**Klf11**	**ENSMUSG00000074170**	**Plekhf1**	**ENSMUSG00000041313**	**Slc7a1**
	**ENSMUSG00000025959**	**Klf7**	**ENSMUSG00000060716**	**Plekhh1**	**ENSMUSG00000032548**	**Slco2a1**
	**ENSMUSG00000019230**	**Lhx9**	**ENSMUSG00000041653**	**Pnpla3**	**ENSMUSG00000037935**	**Smarce1**
	**ENSMUSG00000063804**	**Lin28b**	**ENSMUSG00000049553**	**Polr1a**	**ENSMUSG00000005899**	**Smpd4**
	**ENSMUSG00000053091**	**Lins1**	**ENSMUSG00000026565**	**Pou2f1**	**ENSMUSG00000038145**	**Snrk**
	**ENSMUSG00000027134**	**Lpcat4**	**ENSMUSG00000075028**	**Prdm11**	**ENSMUSG00000051910**	**Sox6**
	**ENSMUSG00000028003**	**Lrat**	**ENSMUSG00000024735**	**Prpf19**	**ENSMUSG00000048562**	**Sp8**
	**ENSMUSG00000024913**	**Lrp5**	**ENSMUSG00000025487**	**Psmd13**	**ENSMUSG00000058297**	**Spock2**
	**ENSMUSG00000008763**	**Man1a2**	**ENSMUSG00000048148**	**Nwd1**	**ENSMUSG00000049506**	**Sppl2c**
	**ENSMUSG00000034751**	**Mast4**	**ENSMUSG00000046610**	**Oacyl**	**ENSMUSG00000026511**	**Srp9**
	**ENSMUSG00000026355**	**Mcm6**	**ENSMUSG00000001173**	**Ocrl**	**ENSMUSG00000042121**	**Ssh1**
	**ENSMUSG00000001419**	**Mef2d**	**ENSMUSG00000072980**	**Oip5**	**ENSMUSG00000037926**	**Ssh2**
	**ENSMUSG00000020818**	**Mfsd11**	**ENSMUSG00000075125**	**Olfr1181**	**ENSMUSG00000026718**	**Stam**
	**ENSMUSG00000032583**	**Mon1a**	**ENSMUSG00000094858**	**Olfr1297**	**ENSMUSG00000049001**	**Ndnf**
	**ENSMUSG00000069769**	**Msi2**	**ENSMUSG00000051706**	**Olfr1325**	**ENSMUSG00000024589**	**Nedd4l**
	**ENSMUSG00000037795**	**N4bp2**	**ENSMUSG00000062314**	**Olfr1505**	**ENSMUSG00000079481**	**Nhsl2**
	**ENSMUSG00000041132**	**N4bp2l1**	**ENSMUSG00000054498**	**Olfr308**	**ENSMUSG00000020902**	**Ntn1**
	**ENSMUSG00000031505**	**Naxd**	**ENSMUSG00000068947**	**Olfr366**	**ENSMUSG00000055254**	**Ntrk2**
	**ENSMUSG00000041120**	**Nbl1**				

TableS2. RNA microarray analysis dataset of KLF4-overexpressing VSMCs vs. control cells

down_KLF4_vs_GFP					
**Gene name**	**Refseq_name**	**GFP count**	**KLF4 count**	**P value**	**Fold Change**
ZNF74	NR_046282	20.22	3.71	<0.001	5.44
ZNF733P	NR_003952	42.06	21.02	0.00746	2.00
ZNF710	NM_198526	25.88	3.71	<0.001	6.96
ZNF101	NM_033204	10.52	1.24	0.00378	8.42
ZBTB20	NM_001164343	10.52	1.24	0.00378	8.42
WWC1	NM_015238	39.64	13.6	<0.001	2.91
VN1R10P	NR_045612	23.46	8.65	0.00775	2.71
TTC23	NM_001288616	23.46	8.65	0.00775	2.71
TSFM	NM_001172697	25.08	7.42	0.00145	3.38
TRIM49B	NM_001206626	10.52	1.24	0.00378	8.42
TNRC6B	NM_001024843	25.08	7.42	0.00145	3.38
TMEM62	NM_001347027	11.32	1.24	0.00225	9.06
TMEM132C	NM_001136103	25.88	3.71	<0.001	6.96
TIAM1	NM_003253	12.13	1.24	0.00134	9.71
TEX14	NM_198393	23.46	8.65	0.00775	2.71
TENM2	NM_001122679	25.08	6.18	<0.001	4.05
TBX19	NM_005149	12.13	1.24	0.00134	9.71
TBC1D21	NM_153356	29.93	4.94	<0.001	6.05
TAPBPL	NR_147129	36.4	4.94	<0.001	7.36
SUMO1P1	NR_002189	42.06	21.02	0.00746	2.00
SS18	NM_001007559	25.08	6.18	<0.001	4.05
SPDYE14P	NR_146072	23.46	8.65	0.00775	2.71
SPATC1	NM_001134374	25.88	3.71	<0.001	6.96
SORCS2	NM_020777	11.32	1.24	0.00225	9.06
SMYD3	NM_001167740	42.06	14.83	<0.001	2.83
SLC39A11	NM_139177	20.22	3.71	<0.001	5.44
SCFD2	NM_152540	28.31	4.94	<0.001	5.72
SAMD4A	NM_015589	20.22	3.71	<0.001	5.44
ROBO3	NM_022370	11.32	1.24	0.00225	9.06
RGS7	NM_001350116	19.41	4.94	0.00244	3.92
PTPN20	NM_001042358	41.25	9.89	<0.001	4.17
PSPC1	NM_001042414	28.31	3.71	<0.001	7.61
PSAPL1	NM_001085382	11.32	1.24	0.00225	9.06
PRUNE2	NM_001330680	42.06	18.54	0.00220	2.27
PPP2R1A	NM_014225	25.08	7.42	0.00145	3.38
PODN	NM_001199081	39.64	13.6	<0.001	2.91
PLD5	NM_001320272	19.41	4.94	0.00244	3.92
PIWIL3	NM_001008496	11.32	1.24	0.00225	9.06

PIP5K1C	NM_001195733	23.46	8.65	0.00775	2.71
PHLPP1	NM_194449	25.88	3.71	<0.001	6.96
PHF2	NM_005392	31.55	4.94	<0.001	6.38
PARVG	NM_022141	20.22	3.71	<0.001	5.44
PARD6G-AS1	NR_028340	37.21	3.71	<0.001	10.01
PAPPA	NM_002581	23.46	8.65	0.00775	2.71
OTUD1	NM_001145373	12.13	2.47	0.00832	4.90
OR11A1	NM_013937	33.97	9.89	<0.001	3.43
OPCML	NM_001319104	45.3	21.02	0.00255	2.15
NINL	NM_001318226	25.08	6.18	<0.001	4.05
NFATC1	NM_172388	28.31	4.94	<0.001	5.72
NEB	NM_004543	28.31	6.18	<0.001	4.58
MYOCD	NM_153604	23.46	8.65	0.00775	2.71
MYO3B	NR_045682	12.13	1.24	0.00134	9.71
MYH9	NM_002473	14.56	2.47	0.00205	5.88
MTFR1L	NM_001099626	22.65	7.42	0.00446	3.05
MN1	NM_002430	21.84	2.47	<0.001	8.81
MIR922	NR_030627	46.11	18.54	<0.001	2.49
MIR548XHG	NR_109925	19.41	4.94	0.00244	3.92
MIR4274	NR_036238	11.32	1.24	0.00225	9.06
MET	NM_001324402	28.31	4.94	<0.001	5.72
MED30	NM_080651	28.31	6.18	<0.001	4.58
ME2	NM_002396	34.78	4.94	<0.001	7.03
MCMBP	NM_001256379	20.22	3.71	<0.001	5.44
MBD2	NM_015832	25.08	4.94	<0.001	5.07
MAPK4	NM_001292039	20.22	3.71	<0.001	5.44
MAFB	NM_005461	46.11	18.54	<0.001	2.49
LOC105378146	NR_136250	28.31	3.71	<0.001	7.61
LOC101929528	NR_105005	25.08	4.94	<0.001	5.07
LOC101927131	NR_110907	33.16	4.94	<0.001	6.70
LOC101927070	NR_126337	61.48	23.49	<0.001	2.62
LOC100507600	NR_045486	30.74	9.89	<0.001	3.11
LMF1	NR_036442	28.31	6.18	<0.001	4.58
LINC02064	NR_104628	19.41	4.94	0.00244	3.92
LINC01173	NR_132376	10.52	1.24	0.00378	8.42
LINC00862	NR_040064	25.08	6.18	<0.001	4.05
LILRB1	NM_001278399	10.52	1.24	0.00378	8.42
LGMN	NM_005606	10.52	1.24	0.00378	8.42
LDLRAP1	NM_015627	24.27	8.65	0.00549	2.80
KLHL11	NM_018143	25.08	4.94	<0.001	5.07
KIDINS220	NM_001348736	31.55	12.36	0.00323	2.55
KCNIP3	NM_013434	10.52	1.24	0.00378	8.42
KAT6B	NM_012330	23.46	8.65	0.00775	2.71
JAG2	NM_145159	23.46	8.65	0.00775	2.71

JADE2	NM_001308143	10.52	1.24	0.00378	8.42
IRF4	NR_046000	41.25	8.65	<0.001	4.76
IFITM10	NM_001170820	20.22	3.71	<0.001	5.44
HULC	NR_004855	25.08	6.18	<0.001	4.05
HOMER2	NM_004839	25.88	3.71	<0.001	6.96
HDAC9	NM_178425	12.13	2.47	0.00832	4.90
GPR12	NM_005288	25.88	3.71	<0.001	6.96
GOSR1	NM_004871	25.08	7.42	0.00145	3.38
GNG4	NM_001098721	12.13	1.24	0.00134	9.71
GAP43	NM_002045	11.32	1.24	0.00225	9.06
GABRA5	NM_000810	12.13	1.24	0.00134	9.71
FHIT	NM_001166243	30.74	9.89	<0.001	3.11
FGF12	NM_004113	10.52	1.24	0.00378	8.42
FER	NM_001308028	25.08	7.42	0.00145	3.38
FENDRR	NR_033925	31.55	4.94	<0.001	6.38
EML4	NM_019063	11.32	1.24	0.00225	9.06
ELOVL5	NM_001301856	31.55	12.36	0.00323	2.55
EEFSEC	NM_021937	42.06	14.83	<0.001	2.83
DYSF	NM_001130986	42.06	14.83	<0.001	2.83
DLGAP2-AS1	NR_103863	20.22	3.71	<0.001	5.44
DHX8	NR_136225	11.32	1.24	0.00225	9.06
DEFB115	NM_001037730	12.13	1.24	0.00134	9.71
CYP27B1	NM_000785	25.08	7.42	0.00145	3.38
CTRB2	NM_001025200	25.08	6.18	<0.001	4.05
CORIN	NM_001278586	25.08	7.42	0.00145	3.38
COL22A1	NM_152888	25.08	7.42	0.00145	3.38
CNTN4	NM_175607	11.32	1.24	0.00225	9.06
CLEC10A	NM_006344	25.08	7.42	0.00145	3.38
CHGA	NM_001275	45.3	21.02	0.00255	2.15
CATSPERE	NM_001130957	16.18	2.47	<0.001	6.53
CASK	NM_003688	10.52	1.24	0.00378	8.42
CACNG4	NM_014405	29.93	4.94	<0.001	6.05
BMPR1B	NM_001256792	23.46	8.65	0.00775	2.71
BDP1	NM_018429	10.52	1.24	0.00378	8.42
BCOR	NM_001123384	33.97	9.89	<0.001	3.43
AVPR1A	NM_000706	39.64	13.6	<0.001	2.91
AOAH-IT1	NR_046764	12.13	1.24	0.00134	9.71
ANK2	NM_001127493	12.13	1.24	0.00134	9.71
AKAP6	NM_004274	20.22	3.71	<0.001	5.44
ADGRB3	NM_001704	29.93	4.94	<0.001	6.05
ADARB2	NM_018702	20.22	3.71	<0.001	5.44
ABTB2	NM_145804	10.52	1.24	0.00378	8.42

up_KLF4_vs_GFP					
Gene name	Refseq_name	GFP count	KLF4 count	P value	Fold Change
ZIC4	NR_040762	10.52	38.32	<0.001	3.64
ZCCHC7	NM_032226	8.09	25.96	0.002	3.21
YIPF4	NM_032312	1.62	16.07	<0.001	9.87
XIRP2	NM_001199144	16.18	35.85	0.006	2.21
WDFY4	NM_020945	5.66	29.67	<0.001	5.23
VSTM5	NM_001144871	8.09	35.85	<0.001	4.43
VLDLR	NM_003383	16.99	53.16	<0.001	3.13
VGLL3	NM_001320493	25.88	51.92	0.003	2.01
TMEM232	NM_001039763	6.47	32.14	<0.001	4.96
TMEM132D-AS2	NR_110058	6.47	30.91	<0.001	4.77
TMEM131	NM_015348	2.43	17.31	<0.001	7.10
TMEM108	NM_023943	8.9	33.38	<0.001	3.75
THSD7B	NM_001316349	5.66	23.49	<0.001	4.14
TCERG1	NM_006706	17.8	46.98	<0.001	2.64
SNORA13	NR_002922	14.56	48.21	<0.001	3.31
SNORA107	NR_132852	23.46	56.87	<0.001	2.42
SHROOM3	NM_020859	4.04	16.07	0.006	3.97
SERPINA7	NM_000354	8.9	35.85	<0.001	4.02
SERF1B	NM_001178087	2.43	23.49	<0.001	9.63
SEMA3D	NM_152754	8.09	25.96	0.002	3.21
RPAP3	NM_024604	1.62	13.6	0.001	8.35
RARB	NM_001290216	16.18	35.85	0.006	2.21
PWRN3	NR_130780	16.18	35.85	0.006	2.21
PRKN	NM_004562	8.09	35.85	<0.001	4.43
PPIG	NM_004792	8.09	25.96	0.002	3.21
PLCH1	NM_001349251	8.09	25.96	0.002	3.21
PFKP	NM_001323069	3.24	13.6	0.009	4.19
PARD3	NM_001184793	2.43	17.31	<0.001	7.10
OXR1	NM_018002	4.85	22.25	<0.001	4.58
OAZ2	NM_002537	8.09	28.43	<0.001	3.51
NXPE3	NM_001134456	8.09	23.49	0.005	2.90
NPY2R	NM_000910	4.04	23.49	<0.001	5.80
NHEJ1	NM_024782	8.9	33.38	<0.001	3.75
MYO3B	NR_045682	8.09	28.43	<0.001	3.51
MTBP	NM_022045	4.85	33.38	<0.001	6.87
MRPS33	NM_016071	4.85	23.49	<0.001	4.84
MRE11	NM_001330347	10.52	38.32	<0.001	3.64
MNDA	NM_002432	4.04	23.49	<0.001	5.80
MFN1	NM_033540	1.62	14.83	<0.001	9.10

MEIKIN	NM_001303622	8.09	25.96	0.002	3.21
MAPK8	NM_001323320	8.09	23.49	0.005	2.90
LUZP2	NM_001009909	2.43	19.78	<0.001	8.11
LOC100129620	NR_033940	18.6	48.21	<0.001	2.59
LINC01721	NR_040102	1.62	13.6	0.001	8.35
LINC01094	NR_038305	8.09	28.43	<0.001	3.51
LINC00443	NR_047026	18.6	48.21	<0.001	2.59
LEUTX	NM_001143832	16.18	35.85	0.006	2.21
LDB2	NM_001304434	4.04	16.07	0.006	3.97
KHDRBS3	NM_006558	8.09	28.43	<0.001	3.51
KCNMA1	NM_001322838	8.9	34.61	<0.001	3.89
KALRN	NM_001322988	8.09	34.61	<0.001	4.27
ITGAV	NM_001144999	5.66	24.72	<0.001	4.36
IDS	NM_000202	4.04	27.2	<0.001	6.72
HSD17B7	NM_016371	4.04	23.49	<0.001	5.80
HS6ST3	NM_153456	1.62	16.07	<0.001	9.87
HMGN5	NM_030763	5.66	29.67	<0.001	5.23
HDHD2	NM_001318765	4.04	16.07	0.006	3.97
GRM8	NM_001127323	8.09	25.96	0.002	3.21
GDA	NR_147240	5.66	24.72	<0.001	4.36
GALNT11	NM_001304514	16.99	39.56	0.002	2.33
GADL1	NM_207359	8.09	23.49	0.005	2.90
FOXP2	NM_148898	5.66	23.49	<0.001	4.14
FBXO10	NM_012166	4.85	23.49	<0.001	4.84
EYS	NM_001142800	8.9	33.38	<0.001	3.75
EPS8	NM_004447	5.66	29.67	<0.001	5.23
EGFEM1P	NR_021485	16.18	35.85	0.006	2.21
EFL1	NM_001322845	4.04	16.07	0.006	3.97
EEPD1	NM_030636	4.04	23.49	<0.001	5.80
DMD	NM_004010	8.09	25.96	0.002	3.21
DMC1	NM_001278208	18.6	53.16	<0.001	2.86
DISC1FP1	NR_104190	12.94	44.5	<0.001	3.44
DDX60	NM_017631	6.47	30.91	<0.001	4.77
DCDC1	NM_020869	13.75	37.09	<0.001	2.70
CSPP1	NM_024790	4.04	16.07	0.006	3.97
CPNE4	NM_130808	8.09	23.49	0.005	2.90
COBL	NM_001346441	2.43	17.31	<0.001	7.10
CNTNAP2	NM_014141	2.43	13.6	0.003	5.58
CFAP47	NM_001304548	3.24	25.96	<0.001	7.99
CDK1	NM_001320918	8.09	24.72	0.003	3.05
CCDC178	NM_198995	16.18	35.85	0.006	2.21
CCDC158	NM_001042784	4.04	16.07	0.006	3.97
CCDC148	NM_001301684	4.04	25.96	<0.001	6.41
CCDC140	NM_153038	23.46	49.45	0.002	2.11

CBWD2	NM_172003	23.46	49.45	0.002	2.11
C4BPB	NM_001017367	4.85	23.49	<0.001	4.84
C14orf37	NM_001320173	4.04	18.54	0.001	4.58
B4GALT6	NM_004775	1.62	16.07	<0.001	9.87
ATXN7L3B	NM_001136262	17.8	46.98	<0.001	2.64
ATP8B4	NR_073597	8.09	25.96	0.002	3.21
ATP7A	NM_000052	6.47	30.91	<0.001	4.77
ANXA2	NM_004039	10.52	27.2	0.006	2.58
ANXA10	NM_007193	4.04	24.72	<0.001	6.11
ANKIB1	NM_019004	2.43	18.54	<0.001	7.60
AGBL4	NM_032785	1.62	13.6	0.001	8.35
ADAM29	NM_001278126	16.18	35.85	0.006	2.21
